# Association of skeletal muscle and serum metabolites with maximum power output gains in response to continuous endurance or high-intensity interval training programs: The TIMES study – A randomized controlled trial

**DOI:** 10.1371/journal.pone.0212115

**Published:** 2019-02-11

**Authors:** Alex Castro, Renata Garbellini Duft, Marina Lívia Venturini Ferreira, André Luís Lugnani de Andrade, Arthur Fernandes Gáspari, Lucas de Marchi Silva, Silas Gabriel de Oliveira-Nunes, Cláudia Regina Cavaglieri, Sujoy Ghosh, Claude Bouchard, Mara Patrícia Traina Chacon- Mikahil

**Affiliations:** 1 Laboratory of Exercise Physiology, School of Physical Education, University of Campinas, Campinas, São Paulo, Brazil; 2 School of Medical Sciences, University of Campinas, Campinas, São Paulo, Brazil; 3 Laboratory of Computational Biology, Pennington Biomedical Research Center, Baton Rouge, Louisiana, United States of America; 4 Cardiovascular & Metabolic Disorders Program and Center for Computational Biology, Duke-NUS Graduate Medical School, Singapore; 5 Human Genomics Laboratory, Pennington Biomedical Research Center, Baton Rouge, Louisiana, United States of America; Victoria University, AUSTRALIA

## Abstract

**Background:**

Recent studies have begun to identify the molecular determinants of inter-individual variability of cardiorespiratory fitness (CRF) in response to exercise training programs. However, we still have an incomplete picture of the molecular mechanisms underlying trainability in response to exercise training.

**Objective:**

We investigated baseline serum and skeletal muscle metabolomics profile and its associations with maximal power output (MPO) gains in response to 8-week of continuous endurance training (ET) and high-intensity interval training (HIIT) programs matched for total units of exercise performed (the TIMES study).

**Methods:**

Eighty healthy sedentary young adult males were randomized to one of three groups and 70 were defined as completers (> 90% of sessions): ET (n = 30), HIIT (n = 30) and control (CO, n = 10). For the CO, participants were asked to not exercise for 8 weeks. Serum and skeletal muscle samples were analyzed by 1H-NMR spectroscopy. The targeted screens yielded 43 serum and 70 muscle reproducible metabolites (intraclass > 0.75; coefficient of variation < 25%). Associations of baseline metabolites with MPO trainability were explored within each training program via three analytical strategies: (1) correlations with gains in MPO; (2) differences between high and low responders to ET and HIIT; and (3) metabolites contributions to the most significant pathways related to gains in MPO. The significance level was set at P < 0.01 or false discovery rate of 0.1.

**Results:**

The exercise programs generated similar gains in MPO (ET = 21.4 ± 8.0%; HIIT = 24.3 ± 8.5%). MPO associated baseline metabolites supported by all three levels of evidence were: serum glycerol, muscle alanine, proline, threonine, creatinine, AMP and pyruvate for ET, and serum lysine, phenylalanine, creatine, and muscle glycolate for HIIT. The most common pathways suggested by the metabolite profiles were aminoacyl-tRNA biosynthesis, and carbohydrate and amino acid metabolism.

**Conclusion:**

We suggest that MPO gains in both programs are potentially associated with metabolites indicative of baseline amino acid and translation processes with additional evidence for carbohydrate metabolism in ET.

## Introduction

Cardiorespiratory fitness (CRF) is commonly assessed by the measurement of maximal oxygen uptake ($\dot{\text{V}}{\text{O}}_{\text{2MAX}}$) or maximum power output (MPO) in incremental exercise tests leading to exhaustion. CRF is the most widely examined human physiological functional capacity [1]. Higher levels of CRF are strongly associated with lower all-cause and cardiovascular disease mortality [2, 3].

Current physical activity guidelines focused on health outcomes recommend that adults engage in continuous endurance exercise at moderate-intensity for ≥ 150 min wk^-1^ or at vigorous-intensity for 75 min wk^-1^ or more [4]. Although it is well known that regular exercise is associated with improvements in CRF [4], there is a large body of animal and human data demonstrating that there is considerable inter-individual variability in CRF response to a standardized dose of exercise [5–9]. Typically, individual responses to standardized continuous endurance training (ET) program have ranged from no gain up to 100% increase in $\dot{\text{V}}{\text{O}}_{\text{2MAX}}$ [5, 7, 10] and to 60% for MPO [11, 12] in groups of previously sedentary heathy adults. Furthermore, inter-individual variability in CRF training responses has also been shown with exposure to high-intensity interval training (HIIT) [13–16]. HIIT is characterized by repeated intense bouts interspersed by short periods of recovery and it generally differs substantially in exercise intensity and metabolic demands from ET [17]. HIIT has been found to produce CRF gains comparable or superior to traditional ET exposure [18]. However, little is known on the relationships between the baseline metabolomics profile and the CRF response patterns to ET or HIIT programs.

It has been previously reported that genomics and muscle transcriptomic profiles were associated with exercise response variability to ET programs [19–21]. In this context, recently, metabolomics has emerged as an “omics” family of technologies with the potential to illuminate biomarkers and metabolic pathways related to complex phenotypes [22, 23]. Metabolomics involves the systematic quantification of small-molecules (metabolites) which provide complementary information to epigenetic events and posttranslational modifications [22]. A metabolite profile (from blood, muscle, adipose tissue, etc) represents a dynamic signature of cellular biochemical activity reflecting the end product of interactions among the genome, transcriptome, proteome and the cellular and tissue environment, which can provide unique insights on phenotypes of interest and their perturbations.

Metabolomics has already been used in a range of exercise studies as recently reviewed [24]. For example, changes in serum and plasma metabolites have been described in response to acute and chronic adaptation to ET or HIIT programs [25–27], as well as differences in metabolite profile in relation to variable cross-sectional CRF levels, but not investigating MPO [28, 29]. To our knowledge, no previous study has investigated the relationships between baseline serum or skeletal muscle metabolites with CRF or MPO trainability. Understanding the association between the baseline metabolite profile and MPO trainability has the potential to generate new biomarkers and clarify the biology of adaptation to regular exercise. In this study, MPO is the dependent variable. MPO as a surrogate of CRF is known to have a high test-retest reliability level and low technical error [11].

Therefore, the present study aimed at investigating baseline serum and skeletal muscle metabolomics profile and related metabolic pathways associated with MPO gains in sedentary young male adults in response to ET and HIIT (TraInability and MEtabolomicS study or TIMES study).

## Methods

### Participants

Young sedentary healthy Caucasian men (18–31 years old) were recruited through local advertisement in Campinas, Brazil to participate in a longitudinal randomized controlled study. Recruitment started in September 2016 and the last post-training assessment was performed in June 2017. Participants were sedentary and did not engage in regular exercise defined as 30 min wk^-1^ at an energy expenditure of 6 METS or more in the previous 4 months. All participants provided a detailed medical history and received a medical examination that included an electrocardiogram at rest. Participants were excluded if they were smoker, hypertensive (blood pressure > 140/90 mm Hg), diabetic (fasting glucose > 7.0 mmol L^-1^), obese (defined as body mass index > 33 kg m^-2^), dyslipidemic (based on medication), or presented evidence of heart diseases, metabolic disorders, significant chronic respiratory conditions or musculoskeletal problems interfering with exercise.

**Fig 1:**
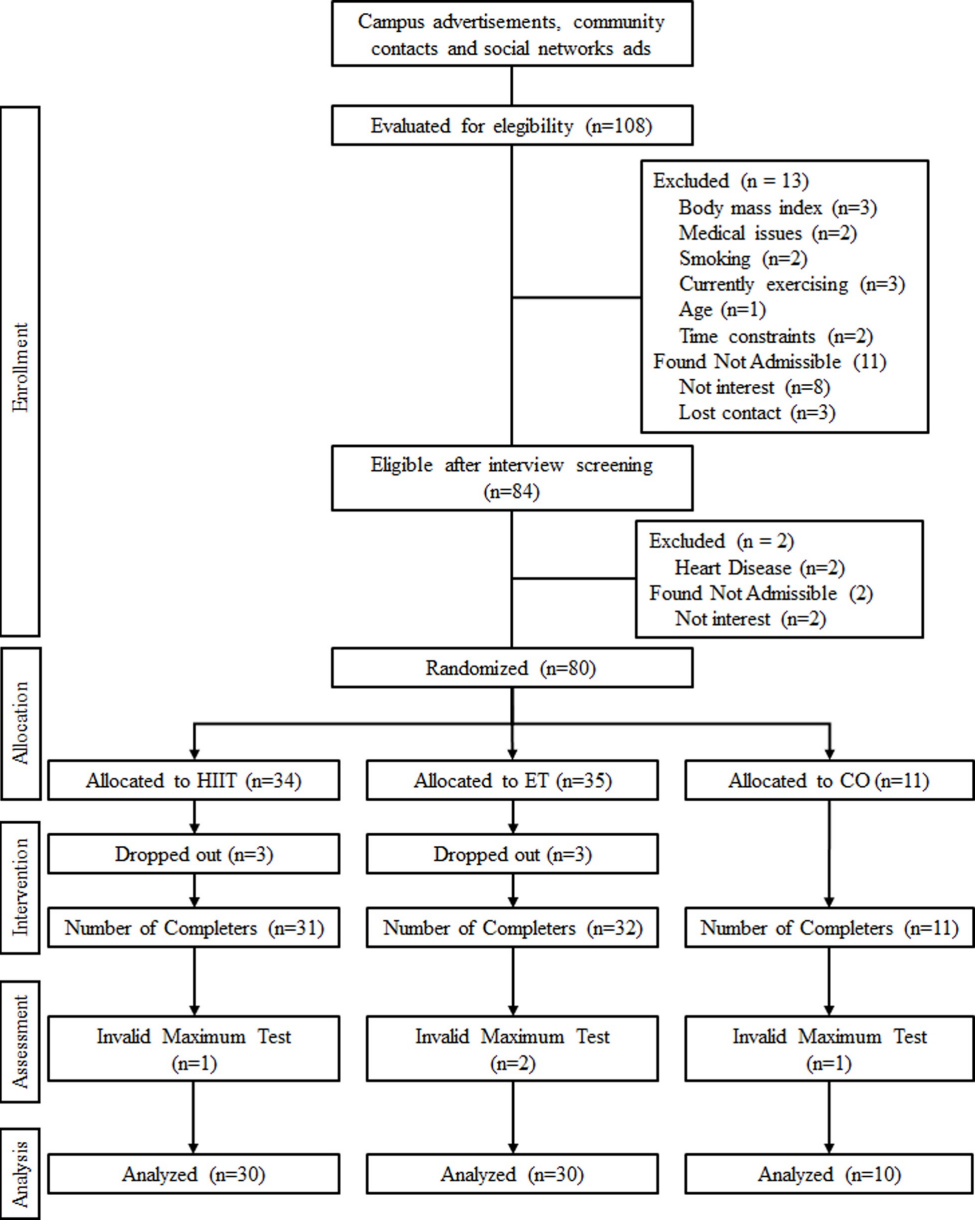
CONSORT flow diagram of participants in the TIMES study.

Initially, a total of 108 young men showed interest and were screened by interview (Fig 1 and S1 Checklist). Of those, 84 underwent a medical examination. Among them, 80 were found to be eligible and were randomized via computer-generated random numbers, after completing all baseline tests, to one of three arms: high-intensity interval training (HIIT; *n =* 34), continuous endurance training (ET; *n =* 35) and control (CO; *n =* 11). An unequal randomization strategy with an approximately 3:1 ratio of subjects between the intervention vs. control arms was used to ensure adequate sample size for correlation analysis and analysis of high and low responders in the intervention groups. Subsequently, 6 participants dropped out early in their treatment arm for personal reasons and 74 completed the intervention, including post-tests. Finally, since the experiment study focuses primarily on mechanisms and is not an effectiveness study, four participants were discarded from analysis due to invalid maximal tests, as objectively determined by failure to attain maximal exertion and associated maximal heart rate. Thus, a total of 70 participants were available for metabolomics explorations.

The sample size was calculated using G*Power 3.2.1 software [30] and determined for MPO in terms of gains expected (~20% or ~50 W) after 8 weeks of intervention, based on data of our laboratory [31], assuming moderate effects of Cohen’s ƒ = 0.3 for within-between interaction design [Group (ET, HIIT and CO) *vs*. Time (Pre and Post intervention)], *r* = 0.5 for correlation design, and type I error of 0.05 for a two side-test to reach a statistical power of at least 80% [32].

The experimental procedures and possible risks associated with the study were explained to all subjects, who provided written informed consent before participation. The study was approved by the Research Ethics Committee from University of Campinas (CAAE: 52997216.8.0000.5404, April 2016) and conducted in conformity with the standards set by the *Declaration of Helsinki* [33]. This study was included in the Brazilian Clinical Trials Registry (ensaiosclinicos.gov.br; RBR-3rh38g; S1 Clinical trial). The study has been registered after enrolment of participants, because trial registration was not required by the ethics committee at the time the trial started. The authors confirm that all ongoing and related trials for this intervention are registered.

### Experimental design

Prior to intervention, resting blood and skeletal muscle biopsy samples were obtained in the fasted state (12 h). After > 72 h, body mass and composition were measured followed by a maximal exercise test on a cycle ergometer designed to measure MPO. The MPO test was repeated 48 h later. About one week later, participants started the eight-week intervention protocol in their assigned arm. Maximal exercise tests were repeated after 4 weeks (to adjust training loads if necessary) and 5 days after the last exercise session. Similarly, in the control group, resting and fasted blood and muscle biopsies were drawn at the beginning of week 8 and the MPO test was performed 3 days later. The MPO and metabolites concentrations were used as our primary and secondary outcomes, respectively.

### Maximal exercise test

Participants were requested to remain sedentary before the MPO test and were instructed to abstain from consuming alcohol (for 48 h), caffeine (for 12 h) and food (for 3 h) before the test. The MPO test and retest was carried out on a cycle ergometer with electromagnetic braking (Corival 400, Lode BV, Groningen, Netherlands). After 5 min of rest on the bike, each participant performed a 3 min warm-up at 50 W followed by workload increments of 25 W every min at a pedaling cadence of 70–80 rpm until exhaustion [34]. Exhaustion was defined as the incapacity to maintain a cadence of at least 70 rpm despite verbal encouragement or being unable to keep with the current workload. Heart rate (HR) was measured during the whole test with a cardiac monitor (S810, Polar, Kepler, Finland) and maximal heart rate (HR_MAX_) was defined as the highest value over 10 s reached at MPO as described below. During the last 15 s of each exercise stage, perceived exertion was recorded with the Borg’s scale [35]. All participants reported ratings of perceived exertion ≥ 17 at end of the test. MPO was calculated as ${\text{W}}_{\text{completed}}+\;[25\cdot \left(\text{t}/60\right)]$, where W_completed_ is the last fully completed workload level and t is the number of valid seconds in the final workload [11]. All tests were performed under controlled conditions of temperature (21–23°C) and relative humidity (50–70%).

The validity of each maximal exercise test was determined based on the achieved HR_MAX_ in relation to the expected HR_MAX_ taking into account all the MPO tests performed by each subject. As reported earlier, the within-subject standard deviation of HR_MAX_ derived from repeated measurements reached about 4 beats min^-1^ [36]. We opted to use 2 x within-subject standard deviation or 8 beats min^-1^ as the cutoff value. Thus, in the case of the pre-training tests, the highest MPO was retained. In the post-training, for the MPO test be valid, the attained HR_MAX_ needed be within 8 beats min^-1^ of the HR_MAX_ associated with the pre-test MPO retained.

### Exercise training

Prior to the main study, a pilot experiment was conducted with 6 subjects who performed the initial ET and then the initial HIIT training session in order to verify their comparability by quantifying the total amount of work performed and the total volume of exercise executed per week in each program. The total amount of work in Joules performed was quite similar between the two exercise sessions (HIIT: 250.2 ± 31.3 J; ET: 250.9 ± 48.5 J) as was the total exercise volume per program calculated as [intensity (% heart rate reserve) x duration (minutes per session) x frequency (sessions per week) x (number of weeks) [37]. The exercise volume is reported in “Units of Exercise” and it reached 816 Units for ET and HIIT, with no difference between the two programs.

The training programs were performed on cycle ergometers, 40 min per session, for 8 weeks. The intensity of training was customized for each individual based on the heart rate reserve (HRR) calculated as the difference between resting and maximum HR values [38]. For ET, participants exercised at 70% HRR for 40 min, three times a week in the first four weeks; and at 75% HRR for 40 min, 4 times a week in the last four weeks. For HIIT, participants exercised at 50% HRR for 5 min, followed by 5 intervals of 4 min at 90% HRR (work phase) interspersed with 3 min at 50% HRR (recovery phase), three days a week, in the first 4 weeks training: and at 60% HRR for 5 min, followed by 5 intervals of 4 min at 90% HRR and 3 min at 60% HRR, 4 days a week in the final four weeks of training. For the CO, participants were asked to not exercise for 8 weeks. Control subjects were contacted after 4 weeks to remind them of the importance of remaining sedentary and to plan for the post program testing visits. Additionally, after four- and eight-weeks, control participants were asked if they have changed their physical activity level. No change involving an increase in walking time greater 30 min a week was recorded.

All exercise sessions were supervised to ensure that the target HR (monitored by the Polar watch and band) and cycling cadence (70–80 rpm) were maintained. The power output of the cycle ergometer was adjusted manually in response to HR variability of each participant at all training sessions. In each exercise session, subjects were required to reach the target HR in less than 90 s. For the first series of exercise, after warming up, we set workload at a power output that elicited 90% of heart rate reserve for HIIT or 70–75% of heart rate reserve for ET, based on cardiorespiratory test. If heart rate appeared to be stable and under the target, additional increments of 10–30 W were applied until the targeted heart rate was reached. For the subsequent sets of exercise sessions, we set the last workload used to reach targeted heart rate, with workload adjustment if heart rate did not meet the target. All subjects trained in target intensity zone in all exercise sessions.

Participants received constant visual feedback on pedal frequency, power output and elapsed time. To minimize possible cardiovascular drift effects due to dehydration and increased body temperature all subjects were encouraged to drink water which was offered *ad libitum* during each exercise session whereas environmental temperature was kept the same throughout all training sessions (21–23°C). There was no airflow provided for cooling.

Participants were asked to remain sedentary outside of the supervised training program, to maintain their current eating patterns, and to report important changes in their daily life routine and their use of new medications during the intervention period. No substantive changes were recorded [i.e.none affecting hours of sleep (± 1 h per day for a week) and walking time (> 30 min at a week)].

### Body composition

Participants were asked to drink only water and not to eat or exercise for 2 h prior to the assessment. Height and body mass were recorded using a stadiometer and digital scale (BOD POD; Cosmed, Chicago, USA) calibrated according to manufacturer guidelines with participants without shoes or outer garments. Next, body density was assessed using air displacement plethysmography (BOD POD Body Composition Tracking System; Cosmed, Chicago, USA) calibrated according to manufacturer guidelines. Body density was converted to body fat percentage using the Siri equation [39].

### Blood and muscle tissue collection

Blood samples and muscle biopsies were collected pre-training between 7:00 and 10:00 am, after 12 h of overnight fast. Participants were instructed to refrain from exercise and alcohol consumption for the preceding 48 h from caffeine consumption for a minimum of 12 h. In preparation for the first laboratory visit, participants consumed a standardized prepacked meal containing 30% of estimated daily energy needs with the following macronutrient composition: 55–60% carbohydrate, 15–20% protein and 20–25% fat. Blood samples were collected from the antecubital vein in serology tubes (Vacuette, 8 ml), centrifuged at 956 *g* for 10 min, and then serum was stored at -80°C until analysis. Muscle biopsies were obtained from the *vastus lateralis* of the dominant leg under local anaesthetic [2–3 ml of 2% lidocaine (Xylestesin)] using the percutaneous biopsy technique combined with suction [40]. Muscle tissue was quickly dissected free from blood and connective tissue, and then samples were immediately frozen in liquid nitrogen and stored at –80°C until further analyses.

### Sample preparation for NMR analysis

Serum samples were centrifuged at 20817 *g* for 45 min at 4°C with a clean 3 kDa membrane centrifuge filter (Amicon Ultra 0.5 ml, Millipore). Filtered serum (250 μL) was diluted in a deuterium oxide solution (D_2_O, 99.9%; Cambridge Isotope Laboratories Inc., USA) containing phosphate buffer (0.1 M, pH 7.4) and 0.5 mM TMSP-d4 (3-(trimethylsilyl)-2,2',3,3'-tetradeuteropropionic acid from Sigma-Aldrich) to a 600 uL final solution, and then transferred to a 5 mm NMR tube (Wilmad Standard Series 5 mm, Sigma-Aldrich) for immediate NMR acquisition [41].

Muscle samples were processed based as described earlier [42, 43]. Briefly, muscle tissue fragments (~40 mg) were weighed, added to a cold methanol/chloroform solution (2:1 v/v, total of 2.5 mL), homogenized on ice (3 times of 30 s each, interspaced by 10 s pause) and sonicated for 3 min with a 10 s pause each minute. A cold chloroform/ultrapure water solution (1:1 v/v, a total of 2.5 mL) was then added to the samples. Samples were briefly vortexed to emulsion and centrifuged (2000 *g*, 30 min, at 4°C). The upper phase containing methanol, water, and polar metabolites was collected and evaporated in a vacuum concentrator (miVac Duo Concentrator, GeneVac, UK). The remaining solid phase was rehydrated in 0.6 mL of D_2_O-containing phosphate buffer (0.1 M, pH 7.4) and 0.5 mM of TMSPd4. Samples were added to a 5-mm NMR tube for immediate scanning.

### NMR data acquisition and metabolite identification

Each spectrum was acquired using a Proton (^1^H) NMR Varian Inova ^1^H NMR spectrometer (Agilent Technologies Inc., Santa Clara, USA) equipped with a triple cold resonance probe operating at a ^1^H resonance frequency of 599.89 MHz and a constant temperature of 298 K (25°C). A total of 256 free induction decays were collected with 32-k data points over a spectral width of 8000 Hz. An acquisition time of 4 s and relaxation delay intervals of 1.5 s were implemented between scans [41]. After all spectra had been acquired, phase adjustment, baseline correction, removal of water signal (4.6–5.1 ppm), spectral calibration and quantification were conducted following the parameters for profiling as defined in Chenomx NMR Suite software 8.31 (Chenomx Inc., Edmonton, Canada) [44]. All spectra were processed with a line broadening (lb) of 0.5 Hz. Metabolites (methanol and ethanol) that were biased due to reagents used in the collection and preparation of samples were not considered for further analysis.

### Determination of technical error of measurement

Only metabolites with satisfactory reproducibility and coefficient of variation (CV) characteristics were retained for the study. In the present research, reproducibility was assessed by the within-subject standard deviation for repeated measures, commonly referred to as the technical error (TE) of measurement [45], and the intraclass correlation (ICC) coefficient. In the case of MPO and HR_MAX_, the values obtained at two tests performed within 48 h at baseline were used for the calculation of TE and CV (*n =* 59). For metabolomics, data obtained on a sample of subjects recruited for the purpose of quantifying TE and CV were used (n *=* 11; age = 22 ± 4 years; BMI = 23 ± 3). The subjects met the same inclusion criteria as the main study. Two blood samples were collected at a 15-min interval in order to quantify the stability of each serum metabolite measurement. Reproducibility and coefficient of variation measurements were not performed for muscle tissue samples.

Briefly, TE was calculated by computing the within-subject standard deviation from repeated measures divided by √2 [46] while CV was derived from TE divided by its measurement mean multiplied by 100 [45]. Reliability of measurement was further examined with the computation of the ICC based on a mixed model analysis of variance [47]. To minimize potential biases due to short-term, unstable metabolites or poorly reproducible assays, we considered for further analysis only serum metabolites that exhibited ICC ≥ 0.75 and CV < 25%.

### Statistical analysis

For all variables, the distributions of scores were checked for major deviation from normality. When appropriate (skewness values > 3.0), logarithmic transformations (log_2_) were used to improve normality of distributions. However, all transformed data are presented herein in their original scale for ease of interpretation.

To compare MPO and other traits as well as metabolite concentrations among the three groups (ET, HIIT and CO) at baseline and their changes with the interventions, we used a one-way ANOVA followed by a Sidak’s adjustment for multiple comparisons. Additionally, a two-way ANOVA (Mixed Linear Model) with a scaled identity covariance matrix structure was used to assess whether there were group (ET, HIIT and CO), time (Pre- and Post-training) and interaction group*time effects with time as a repeated measure effect, and assuming subjects as a random factor. Whenever a significant F-value was obtained, Sidak’s adjustment was performed to verify where the differences reside. In another series of comparisons, the lowest (1^st^; low responders—LRE) and highest (3^rd^; high responders—HRE) tertiles of gains in MPO in response to ET and HIIT were compared for baseline metabolite concentrations using a Student unpaired t test. To identify baseline metabolites and other traits associated with MPO, reproducible serum and skeletal muscle metabolites were correlated to gains in MPO (W) in response to each intervention program (ET and HIIT) using Pearson correlation coefficient. These analyses were carried out using the PASW statistics software version 18.0 (SPSS, Chicago, IL).

For this exploratory and hypothesis-generating study, a large number of statistical tests were performed. We therefore adjusted the significance level threshold at a nominal value of *P* < 0.01, recognizing that a full Bonferroni adjustment is likely to be too conservative, leading to reduced discovery from greater number of false negative observations. To supplement the above, we calculated the 95% confidence intervals of the effect size (ES: mean difference divided by pooled SD from all subjects) of each baseline metabolite concentration between LRE and HRE. If the confidence intervals did not cross zero, the difference was considered significant [48].

To identify the most relevant baseline metabolic pathways related to MPO gains for each program, the set of serum and skeletal muscle metabolites that had correlation coefficients of 0.2 and above (separately in ET and HIIT) were retained for pathway over-representation and pathway topology analyses using the web-based tool MetaboAnalyst 4.0 (http://www.metaboanalyst.ca). The pathway analysis was based on ‘Homo Sapiens’ library using Hypergeometric Test for Over Representation Analysis and Relative-Betweeness Centrality for Test Pathway Topology Analysis [49]. For the pathway enrichment analysis, we used a false discovery rate of 0.1 [50] to account for multiple tests while allowing for hypothesis generation to provide good balance between controlling for false positives and being able to identify true effects [51]. The threshold for correlation coefficient was selected on the basis of the range of effects expected for molecular predictors of CRF trainability (0.07 ≤ r ≤ 0.26) as observed in previous studies [19, 20].

Finally, the baseline metabolites most associated with MPO trainability were those that scored highly among the three levels of evidences used in the present analytical strategy: (1) correlations with gains in MPO (r ≥ 0.2); (2) differences between LRE and HRE; and (3) metabolites contributions to the most significant pathways related to gains in MPO.

## Results

### Reproducibility analysis

Both MPO and HR_MAX_ showed excellent reproducibility in the test-retest (48 h) situation, with CVs lower than 3% and ICCs higher than 0.95 (Table 1). Furthermore, for the 52 serum metabolites tested in a related pilot study of TIMES with blood samples obtained 15-min apart, ICCs and CVs were in a range of 0.79 to 1.00 and 3–23%, respectively, for all but 9 metabolites (*2-Aminobutyrate*, *2-Hydroxybutyrate*, *2-Oxoglutarate*, *Acetate*, *Acetoacetate*, *Fumarate*, *Glucose*, *Methylamine and Oxypurinol*) which had ICCs below 0.75 and CVs above 25% (S1 Table). These latter metabolites were judged to be of low reproducibility and not considered for further analysis.

**Table 1:** Reproducibility of MPO and HR_MAX_ based on test-retest within 48 h (*n* = 59) in TIMES.

Variables	Mean		SD	TE	CV%	ICC
MPO (W)	236.9	±	36.2	6.54	2.8	0.98
HR_MAX_ (bpm min^-1^)	192	±	9	2.85	1.5	0.95

Mean and standard deviation (SD) were calculated from the data of both tests. MPO: Maximal power output; HR_MAX_: Maximal heart rate; TE: Technical error defined as the within-subject standard deviation calculated from repeated measurements; CV: Coefficient of variation derived from the technical error and the measurement mean, expressed as a percentage; ICC: Intraclass correlation coefficient.

### Baseline characteristics

The ET, HIIT and CO groups were comparable for age, height, body mass, body fat percentage, body mass index (BMI), fasting glucose, systolic blood pressure, diastolic blood pressure, HR at rest, HR_MAX_ and estimated cardiorespiratory fitness in METS (Table 2), MPO (Table 3), serum metabolite concentration levels (S2 Table) and skeletal muscle metabolite concentrations (S3 Table) at baseline.

**Table 2:** Baseline characteristics of subjects in TIMES.

Variables	ET (*n* = 30)			HIIT (*n* = 30)			CO (*n* = 10)		
	Mean	±	SD	Mean	±	SD	Mean	±	SD
Age (years)	23.3	±	3.4	23.5	±	2.6	23.6	±	3.5
Height (m)	1.7	±	0.1	1.7	±	0.1	1.7	±	0.0
Body mass (kg)	72.1	±	12.1	72.1	±	10.3	76.5	±	8.4
Body fat percentage (%)	20.3	±	7.3	21.3	±	7.6	21.6	±	5.7
BMI (kg m^2^)	23.9	±	3.4	23.8	±	2.7	25.0	±	2.6
Fasting glucose (mmol L^-1^)^a^	4.0	±	0.4	4.2	±	0.6	3.9	±	0.3
Systolic BP (mm Hg)	114.1	±	13.1	116.1	±	11.2	118.7	±	11.0
Diastolic BP (mm Hg)	71.8	±	10.1	74.2	±	9.3	71.2	±	10.5
HR at rest (beats min^-1^)	71	±	9	70	±	7	71	±	9
HR_MAX_ (beats min^-1^)	192	±	9	192	±	8	195	±	9
Cardiorespiratory fitness (METS)	12.3	±	1.9	12.3	±	1.8	12.1	±	1.1

ET: Continuous endurance training; HIIT: High-intensity interval training; CO: Control; BMI: Body mass index; BP: Blood pressure; HR: Heart rate; HR_MAX_: Maximal heart rate.

^a^ Serum glucose was obtained from the metabolomics assay; at posteriori, no subject exhibited a serum glucose > 7 mmol L^-1^ at baseline. There were no significant differences between groups for any variables (*P* > 0.01 for all one-way ANOVA tests).

**Table 3:** MPO and HR_MAX_ pre and post-intervention in TIMES.

Variables		ET (*n =* 30)			HIIT (*n =* 30)			CO (*n =* 10)		
		Mean	±	SD	Mean	±	SD	Mean	±	SD
MPO (W)^a^^b^^c^	Pre	237.5	±	38.4	237.6	±	32.4	249.5	±	28.7
	Post	286.5	±	37.6*†	294.2	±	35.3*†	239.5	±	26.3
∆ MPO (W)^a^		49.0	±	15.3†	56.6	±	17.2†	-10.0	±	9.3
∆ MPO (%)^a^		21.4	±	8.0†	24.3	±	8.5†	-3.9	±	3.4
HR_MAX_ (beats min^-1^)^b^	Pre	192	±	9	192	±	8	195	±	9
	Post	192	±	7	191	±	7	195	±	12
∆ HR_MAX_ (beats min^-1^)^a^		0	±	5	-1	±	4	0	±	5
∆ HR_MAX_ (%)^a^		-0.1	±	2.6	-0.3	±	2.2	-0.1	±	2.7

ET: Continuous endurance training; HIIT: High-intensity interval training; CO: Control; MPO: Maximal power output; HR_MAX_: Maximal heart rate; ∆: Change pre to post-intervention.

^a^ Baseline values and changes were analysed by one-way ANOVA.

^b^ Interactions group*time were analysed by two-way ANOVA (Mixed Linear Model);

^c^ Significant interaction group*time.

* Difference from pre (P < 0.01).

^†^ Difference from CO group (P < 0.01).

### Intervention effects

Adherence to training was similar between ET and HIIT programs (97.6 ± 3.0% *vs*. 97.3 ± 3.5% of training sessions completed, respectively, *P* = 0.681). There was no difference between the two training programs for the total amount of work performed on the cycle ergometer across all sessions (*P* > 0.01). The total amount of work performed in each training program was as follows: 8711.7 ± 1740.9 kJ for ET and 7667.0 ± 1402.7 for HIIT.

The main gain in MPO in response to ET reached 49.9 ± 15.3 W (21.4 ± 8.0%) and to HIIT 56.6 ± 17.2 W (24.3 ± 8.5%) with wide ranges from 20 to 77 W (8.3 to 39.3%) for ET and from 31 to 94 W (12.9 to 44.6%) for HIIT (Table 3 and S1 Fig). There was a significant group*time interaction effect (*P* < 0.001), where ET and HIIT groups increased MPO similarly from baseline to post-training (*P* < 0.001 for both) while the CO group experienced no change in MPO (*P* > 0.01). There were no significant differences in absolute (W), and relative (%) gains in MPO between ET and HIIT programs, and both exercise programs registered higher gains in MPO compared to CO group (P < 0.001 for all comparisons). There were no significant interactions or main effects for HR_MAX_ (Table 3). Additionally, no cardiovascular drift across the duration of training sessions were observed for either training program (S2 Fig).

### Associations between baseline values and MPO gains

There were no significant correlations between baseline characteristics (age, body mass, body fat percentage, BMI, and MPO) and MPO gains with ET and HIIT programs (*P* > 0.01 for all, Table 4). In the ET program, baseline serum metabolite concentration levels that were correlated with the gains in MPO at *r* ≥ 0.2 and better included were o-acetylcarnitine, 3-hydroxybutyrate, propyleneglycol and others as summarized in Table 5. In the case of baseline skeletal muscle metabolites, alanine, glutamate, histidine, phenylalanine, proline, threonine, creatinine, glutathione, isobutyrate, 3-methylxanthine, AMP, 2-phosphoglycerate, histamine and pyruvate concentrations among others were correlated with the ET gains in MPO (Table 6). In the HIIT program, the most correlated serum metabolites included lysine, asparagine, and tyrosine (Table 5), while baseline skeletal muscle *τ*-methylhistidine and glycolate were the best correlates of the gains in MPO (Table 6).

**Table 4:** Correlation coefficients (*r*) between gains (∆) in MPO (W) and baseline characteristics.

Variables	ET (*n =* 30)	HIIT (*n =* 30)
	∆ MPO (W)	∆ MPO (W)
MPO (W)	-0.255	-0.091
Age (years)	-0.226	0.061
Body mass (kg)	-0.018	-0.090
Fat mass (%)	-0.166	-0.078
BMI (kg m^2^)	-0.183	-0.191

ET: Continuous endurance training; HIIT: High-intensity interval training; CO: Control; MPO: Maximal power output. BMI: Body mass index. No correlation reached statistical significance (P > 0.01).

**Table 5:** Pearson correlation coefficients (*r*) between gains (∆) in MPO (W) and baseline serum metabolites concentration levels in TIMES.

Serum metabolites^#^	ET (*n =* 30)		HIIT (*n =* 29)
	∆ MPO		∆ MPO
***Amino acids***			
Alanine	-0.02		-0.09
Asparagine	**0.36**		**-0.33**
Glutamine	**0.28**		-0.11
Glycine	0.15		0.02
Histidine	0.13		**-0.20**
Isoleucine	0.01		0.12
Lysine	-0.08		**-0.36**
Methionine	**0.24**		**-0.21**
Phenylalanine	0.18		**-0.29**
Proline	-0.05		0.12
Threonine	**0.29**		0.02
Tyrosine	0.05		**-0.35**
Valine	0.09	^LT^	0.03
***Carboxylic acids***			
Betaine	-0.13		-0.10
Creatinine	**0.22**		-0.19
Guanidoacetate	0.15		**0.23**
N,N-Dimethylglycine	-0.14		**-0.22**
Ornithine	**0.26**		0.01
Succinate	0.14		-0.13
Creatine	-0.06		**0.21**
Creatinephosphate	-0.01		**0.25**
Formate	-0.19		-0.09
***Fatty acids***			
2-Hydroxyisocaproate	0.16	^LT^	0.18
2-Hydroxyisovalerate	0.05		-0.05
Methylsuccinate	**0.21**		-0.03
O-Acetylcarnitine	**0.42**		0.11
***Hydroxy acids***			
3-Hydroxybutyrate	**0.42**		**0.24**
Lactate	0.11		**-0.22**
Glycolate	**0.21**		0.07
***Imidazopyrimidines***			
Hypoxanthine	**0.21**		-0.17
Xanthine	-0.02		-0.10
***Organic carbonic acids***			
N-Methylhydantoin	-0.17		**0.23**
Urea	-0.08		**0.27**
***Organic oxygen compounds***			
Glycerol	**0.33**		**-0.20**
Carnitine	0.15		0.06
Choline	0.06		-0.10
Citrate	0.18		-0.11
Dimethyl-sulfone	-0.01		0.13
Trimethylamine	0.12		**-0.21**
Propyleneglycol	**0.42**		-0.07
***Unclustered***			
Dimethylamine	0.15		**-0.21**
Inosine	-0.05		-0.02
Pyruvate	-0.02		-0.10

ET: Continuous endurance training; HIIT: High-intensity interval training; MPO: Maximal power output; ∆: Change pre to post intervention. ^LT^ Data log transformed before analysis. Bold values are correlation coefficients (*r*) ≥ 0.2.

^#^ Metabolites chemical taxonomy was based on class and sub-class from Human Metabolome Database.

**Table 6:** Pearson correlation coefficients (*r*) between gains in MPO and baseline muscle metabolites levels in TIMES.

Skeletal muscle metabolites^#^	ET (*n =* 29)		HIIT (*n =* 28)	
	∆ MPO		∆ MPO	
***Alcohols and polyols***				
Ethyleneglycol	0.19		-0.06	
Myo-Inositol	**0.24**		-0.04	
***Amino acids***				
Alanine	**0.40**	^LT^	0.01	
Anserine	0.18	^LT^	**-0.24**	
β-Alanine	**0.20**	^LT^	-0.02	
Glutamate	**0.40**	^LT^	0.05	
Glutamine	**0.34**	^LT^	-0.04	
Glycine	**0.36**	^LT^	-0.08	
Histidine	**0.40**	^LT^	-0.06	
Isoleucine	**0.30**		0.17	
Leucine	0.13	^LT^	**0.20**	
Phenylalanine	**0.46**		0.00	
Proline	**0.38**	^LT^	0.19	
Threonine	**0.46**	^LT^	-0.12	
Tyrosine	0.12	^LT^	0.11	
Valine	-0.12		0.11	
***Carboxylic acids***				
Acetate	**0.35**		0.14	
Betaine	0.07		-0.16	
Citrate	0.08		-0.01	
Creatine	**0.35**	^LT^	0.13	
Creatinephosphate	-0.04	^LT^	-0.14	
Creatinine	**0.38**		0.02	
Formate	**0.23**	^LT^	0.06	
Fumarate	**0.21**		0.05	
Glutathione	**0.40**	^LT^	**-0.25**	
Isobutyrate	**0.45**	^LT^	0.09	
Isocitrate	**0.26**		**-0.29**	
Maleate	0.01		0.10	
Malonate	0.19	^LT^	0.09	
N,N-Dimethylglycine	**0.33**		-0.12	
N-Acetylaspartate	**0.33**		**-0.20**	
N-Acetylglutamine	**0.23**		-0.19	
Nicotinurate	0.18		0.00	
Ornithine	**0.30**	^LT^	-0.19	
Succinate	**0.20**	^LT^	0.15	
π-Methylhistidine	**0.21**	^LT^	0.04	
*τ*-Methylhistidine	**-0.28**		**0.35**	^LT^
***Fatty acids***				
2-Hydroxyisocaproate	**0.34**	^LT^	-0.04	
3-Hydroxyisovalerate	**0.33**	^LT^	**0.23**	
O-Acetylcarnitine	**0.26**	^LT^	0.12	
***Hydroxy acids***				
Glycolate	-0.09		**-0.42**	
Lactate	**0.30**		0.04	
***Imidazopyrimidines***				
3-Methylxanthine	**0.38**		**-0.25**	
Oxypurinol	**0.26**	^LT^	-0.06	
Theophylline	**0.36**		-0.15	
***Nucleosides and nucleotides***				
ADP	**0.31**		-0.01	
AMP	**0.38**		-0.04	
ATP	**0.28**	^LT^	-0.03	
NAD+	**0.23**	^LT^	-0.16	
NADP+	0.11	^LT^	**-0.23**	
***Organic oxygen compounds***				
2-Phosphoglycerate	**0.37**		0.15	
Glucose	**0.22**	^LT^	-0.19	
Glycerol	0.01		0.08	
***Organic nitrogen compounds***				
Carnitine	**0.29**		0.02	
Choline	-0.16		-0.12	
Dimethylamine	**0.36**	^LT^	0.05	
Histamine	**0.45**	^LT^	0.13	
Methylamine	**0.29**	^LT^	**0.30**	
N-Nitrosodimethylamine	**0.22**		-0.02	
Trimethylamine	0.17	^LT^	-0.15	
Trimethylamine-N-oxide	**0.31**	^LT^	-0.15	
Tartrate	-0.04		0.04	
***Unclustered***				
2-Hydroxyphenylacetate	**0.36**		0.07	
Acetamide	0.16		**-0.30**	
Carnosine	0.11		-0.06	
Dimethylsulfone	**0.24**		**-0.24**	
Niacinamide	**0.32**		-0.01	
Pyrimidine	0.11	^LT^	0.04	
Pyruvate	**0.42**		-0.18	
Taurine	0.18	^LT^	-0.03	

ET: Continuous endurance training; HIIT: High-intensity interval training; MPO: Maximal power output; ∆: Change pre to post intervention. ^LT^ Data log transformed before analysis. Bold values are correlation coefficients (*r*) ≥ 0.2.

^#^ Metabolites chemical taxonomy was based on class and sub-class from Human Metabolome Database.

### Differences between high and low responders (HRE and LRE)

There were no significant baseline differences between HRE (upper tertile) and LRE (lower tertile) in ET and HIIT programs for age, height, body mass, body fat percentage, BMI, HR_MAX_ and MPO (*P* > 0.01 for all, S4 Table). As expected, in both exercise programs, HRE presented higher absolute and relative MPO gains compared to LRE (*P* < 0.0001 for both).

For ET program, HRE presented higher baseline serum concentration levels than LRE for 3-hydroxybutyrate (*P* = 0.062), glycerol (*P =* 0.035), methylsuccinate (*P =* 0.041), O-acetylcarnitine (*P =* 0.019) as revealed by the pattern of findings summarized as effect sizes and 95% confidence intervals in Table 7. For skeletal muscle metabolites, higher concentration levels were observed in HRE for alanine (*P =* 0.049), AMP (*P =* 0.051), creatinine (*P =* 0.035), proline (*P =* 0.041), pyruvate (*P =* 0.035), threonine (*P =* 0.044), but lower concentrations of isobutyrate (*P =* 0.044) when compared to LRE (Table 8). In the case of the HIIT program, HRE subjects showed higher baseline serum concentrations of creatine (*P =* 0.049) and lower concentration levels of lysine (*P =* 0.054), and phenylalanine (*P =* 0.048) compared to LRE (Table 7). As for skeletal muscle metabolites, HRE displayed lower concentrations of glycolate compared to LRE (Table 8).

**Table 7:** Baseline differences in serum metabolites concentration levels between low responders (LRE) and high responders (HRE) to ET and HIIT programs in TIMES. Data are mean ± standard deviation, plus confidence intervals of the effect size (ES) of differences.

Serum metabolites (mM)^#^	ET						ES	95% CI		Serum metabolites (mM)^#^	HIIT						ES	95% CI	
	LRE (*n =* 10)			HRE (*n =* 10)							LRE (*n =* 9)			HRE (*n =* 10)					
3-Hydroxybutyrate	0.0364	±	0.0337	0.1097	±	0.1063*	**-0.93**	**-1.85**	**-0.01**	3-Hydroxybutyrate	0.0830	±	0.0641	0.1169	±	0.1648	-0.27	-1.17	0.64
Asparagine	0.0333	±	0.0109	0.0414	±	0.0143	-0.64	-1.54	0.26	Asparagine	0.0402	±	0.0115	0.0336	±	0.0125	0.55	-0.37	1.47
Creatinine	0.0658	±	0.0147	0.0783	±	0.0217	-0.67	-1.57	0.23	Creatine	0.0091	±	0.0072	0.0180	±	0.0105*	**-0.97**	**-1.93**	**-0.02**
Glutamine	0.3342	±	0.0648	0.4093	±	0.1909	-0.53	-1.42	0.37	Creatinephosphate	0.0033	±	0.0020	0.0049	±	0.0029	-0.65	-1.58	0.27
Glycerol	0.1797	±	0.0382	0.2352	±	0.0670*	**-1.02**	**-1.95**	**-0.09**	Dimethylamine	0.0055	±	0.0041	0.0044	±	0.0043	0.26	-0.64	1.17
Glycolate	0.0146	±	0.0020	0.0174	±	0.0069	-0.54	-1.44	0.35	Guanidoacetate	0.0311	±	0.0191	0.0456	±	0.0203	-0.73	-1.66	0.20
Hypoxanthine	0.0031	±	0.0007	0.0037	±	0.0011	-0.56	-1.46	0.33	Glycerol	0.2253	±	0.0679	0.1838	±	0.0665	0.62	-0.30	1.54
Methionine	0.0223	±	0.0061	0.0247	±	0.0064	-0.39	-1.28	0.49	Histidine	0.0950	±	0.0166	0.0876	±	0.0106	0.54	-0.38	1.46
Methylsuccinate	0.0107	±	0.0046	0.0145	±	0.0030*	**-0.99**	**-1.92**	**-0.06**	Lactate	1.9549	±	0.5674	1.6320	±	0.3145	0.71	-0.21	1.64
O-Acetylcarnitine	0.0040	±	0.0025	0.0073	±	0.0031*	**-1.16**	**-2.11**	**-0.22**	Lysine	0.1066	±	0.0239	0.0832	±	0.0252*	**0.95**	**0.00**	**1.90**
Ornithine	0.0248	±	0.0095	0.0350	±	0.0170	-0.74	-1.65	0.17	Methionine	0.0212	±	0.0092	0.0181	±	0.0049	0.43	-0.48	1.34
Propylene glycol	0.0143	±	0.0047	0.0188	±	0.0049	-0.92	-1.85	0.00	N.N-Dimethylglycine	0.0032	±	0.0005	0.0028	±	0.0007	0.57	-0.35	1.49
Threonine	0.1164	±	0.0140	0.1371	±	0.0450	-0.62	-1.52	0.28	N-Methylhydantoin	0.0012	±	0.0005	0.0016	±	0.0008	-0.66	-1.59	0.26
										Phenylalanine	0.0651	±	0.0146	0.0545	±	0.0058*	**0.98**	**0.03**	**1.93**
										Trimethylamine	0.0020	±	0.0007	0.0018	±	0.0006	0.32	-0.59	1.22
										Tyrosine	0.0758	±	0.0164	0.0640	±	0.0079	0.94	-0.01	1.89
										Urea	0.5102	±	0.1680	0.6089	±	0.2848	-0.42	-1.33	0.49

ET: Continuous endurance training; HIIT: High-intensity interval training; LRE and HRE were stratified by the 1^st^ and 3^rd^ tertiles, respectively, from the gains in maximal power output (MPO) in response to ET and HIIT. ES: Effect size calculated as the standardized difference between HRE and LRE in standard deviation units; CI: Confidence intervals.

^#^ Set of metabolites with correlation coefficients (*r*) between baseline values and (MPO) gains higher than 0.2.

*Difference from LRE (as determined by the 95% CI for ES that did not cross zero), bold values.

**Table 8:** Baseline differences in skeletal muscle metabolites concentration levels between low responders (LRE) and high responders (HRE) to ET and HIIT programs at baseline in TIMES. Data are mean ± standard deviation.

Skeletal muscle metabolites (mM g^-1^)	ET						ES	95% CI		Skeletal muscle metabolites (mM.g^-1^)	HIIT						ES	95% CI	
	LRE (*n =* 10)			HRE (*n =* 10)							LRE (*n =* 9)			HRE (*n =* 10)					
2-Hydroxyisocaproate^LT^	0.0333	±	0.0174	0.1208	±	0.2262	-0.64	-1.54	0.26	3-Hydroxyisovalerate	0.0916	±	0.0224	0.1217	±	0.0574	-0.68	-1.60	0.25
2-Hydroxyphenylacetate	0.0467	±	0.0229	0.0761	±	0.0596	-0.62	-1.52	0.28	3-Methylxanthine	0.0820	±	0.0465	0.0570	±	0.0262	0.67	-0.26	1.60
2-Phosphoglycerate	1.1580	±	0.8462	3.0567	±	3.4077	-0.76	-1.67	0.14	Acetamide	0.0515	±	0.0181	0.0336	±	0.0211	0.90	-0.04	1.85
3-Hydroxyisovalerate^LT^	0.0911	±	0.0210	0.2880	±	0.5351	-0.52	-1.41	0.37	Anserine	0.0819	±	0.0621	0.0544	±	0.0401	0.53	-0.38	1.45
3-Methylxanthine	0.0642	±	0.0424	0.1111	±	0.0707	-0.81	-1.72	0.11	Dimethylsulfone	0.0218	±	0.0107	0.0147	±	0.0045	0.88	-0.06	1.82
Acetate	0.3637	±	0.1309	0.6021	±	0.4515	-0.72	-1.62	0.19	Glutathione	0.1651	±	0.0822	0.1241	±	0.0766	0.52	-0.40	1.43
ADP	0.0203	±	0.0144	0.0319	±	0.0259	-0.56	-1.45	0.34	Glycolate	0.8198	±	0.3807	0.4438	±	0.4032*	**0.96**	**0.01**	**1.91**
Alanine^LT^	1.5874	±	0.4245	2.7646	±	2.1839*	**-0.95**	**-1.87**	**-0.02**	Isocitrate	0.2628	±	0.1744	0.1488	±	0.0635	0.89	-0.06	1.83
AMP	0.0608	±	0.0416	0.1152	±	0.0711*	**-0.93**	**-1.86**	**-0.01**	Leucine	0.1394	±	0.0866	0.1817	±	0.1195	-0.40	-1.31	0.51
ATPLT	0.0526	±	0.0246	0.0914	±	0.1154	-0.50	-1.38	0.39	Methylamine	0.0568	±	0.0251	0.0757	±	0.0592	-0.41	-1.32	0.50
β-Alanine^LT^	0.1182	±	0.0655	0.2497	±	0.4442	-0.26	-1.14	0.62	N.N-Dimethylglycine	0.036	±	0.014	0.031	±	0.0135	0.34	-0.56	1.25
Carnitine	1.4222	±	1.0910	2.0486	±	1.1493	-0.56	-1.45	0.33	NADP+	0.0188	±	0.0083	0.0139	±	0.0114	0.49	-0.42	1.41
Creatine^LT^	12.587	±	3.6706	22.869	±	22.243	-0.78	-1.68	0.25	τ-Methylhistidine	0.4473	±	0.3936	0.9128	±	1.1093	-0.55	-1.46	0.37
Creatinine	0.1051	±	0.0587	0.2069	±	0.1287*	**-1.02**	**-1.95**	**-0.09**										
Dimethylamine^LT^	0.0197	±	0.0113	0.0506	±	0.0801	-0.72	-1.62	0.35										
Dimethylsulfone	0.0193	±	0.0079	0.0262	±	0.0204	-0.45	-1.33	0.44										
Formate^LT^	0.8568	±	0.5706	1.3886	±	1.3546	-0.50	-1.39	0.38										
Fumarate	0.0442	±	0.0134	0.0574	±	0.0412	-0.43	-1.32	0.46										
Glucose^LT^	0.6620	±	0.3043	0.8370	±	0.7496	-0.18	-1.06	0.70										
Glutamate^LT^	0.6019	±	0.3278	1.2540	±	1.8610	-0.69	-1.59	0.21										
Glutamine^LT^	6.4535	±	2.1208	11.689	±	11.399	-0.88	-1.80	0.04										
Glutathione^LT^	0.0807	±	0.0464	0.1832	±	0.2377	-0.72	-1.62	0.19										
Glycine^LT^	0.5711	±	0.2411	1.0937	±	1.0838	-0.90	-1.82	0.02										
Histamine^LT^	0.0956	±	0.0739	0.3303	±	0.5266	-0.70	-1.60	0.20										
Histidine^LT^	0.2220	±	0.0991	0.5340	±	0.5839	-0.86	-1.77	0.06										
Isobutyrate^LT^	0.0373	±	0.0137	0.2084	±	0.2820*	**-1.02**	**-1.95**	**-0.09**										
Isocitrate	0.1945	±	0.1482	0.3513	±	0.2428	-0.78	-1.69	0.13										
Isoleucine	0.1003	±	0.0439	0.2211	±	0.2751	-0.61	-1.51	0.28										
Lactate	3.5887	±	0.8784	4.8606	±	3.0539	-0.57	-1.46	0.33										
Methylamine^LT^	0.0473	±	0.0478	0.0973	±	0.1462	-0.68	-1.58	0.22										
Myo-Inositol	0.5446	±	0.3353	0.7324	±	0.7103	-0.34	-1.22	0.54										
N,N-Dimethylglycine	0.0287	±	0.0177	0.0431	±	0.0341	-0.53	-1.42	0.36										
N-Acetylaspartate	0.0570	±	0.0233	0.0684	±	0.0233	-0.49	-1.38	0.40										
N-Acetylglutamine	0.0465	±	0.0212	0.0539	±	0.0213	-0.35	-1.23	0.53										
NAD+^LT^	0.1206	±	0.0631	0.1676	±	0.1685	-0.37	-1.25	0.51										
Niacinamide	0.1062	±	0.0626	0.1883	±	0.1918	-0.58	-1.47	0.32										
N-Nitrosodimethylamine	0.0624	±	0.0151	0.0730	±	0.0494	-0.29	-1.17	0.59										
O-Acetylcarnitine^LT^	0.5165	±	0.3459	0.8350	±	1.2147	-0.26	-1.14	0.62										
Ornithine^LT^	0.1196	±	0.0275	0.2086	±	0.2090	-0.39	-1.27	0.50										
Oxypurinol	0.6912	±	0.5124	1.3503	±	1.8772	-0.48	-1.37	0.41										
Phenylalanine	0.0698	±	0.0154	0.1372	±	0.1236	-0.76	-1.67	0.14										
π-Methyhistidine^LT^	0.1140	±	0.1634	0.1388	±	0.2073	-0.34	-1.23	0.54										
Proline^LT^	0.2829	±	0.1480	1.1477	±	2.1528*	**-0.98**	**-1.91**	**-0.06**										
Pyruvate	0.1137	±	0.0482	0.2426	±	0.1721*	**-1.02**	**-1.95**	**-0.09**										
Succinate^LT^	0.0816	±	0.0169	0.1120	±	0.0973	-0.38	-1.26	0.51										
Theophylline	0.1515	±	0.0958	0.2477	±	0.2231	-0.56	-1.45	0.33										
Threonine^LT^	0.2113	±	0.0911	0.4581	±	0.5168*	**-0.97**	**-1.89**	**-0.04**										
Trimethylamine-N-oxide^LT^	0.1011	±	0.0990	0.2611	±	0.3509	-0.73	-1.63	0.18										
τ-Methylhistidine	0.5810	±	0.4570	0.3394	±	0.3076	0.62	-0.28	1.52										

ET: Continuous endurance training; HIIT: High-intensity interval training; LRE and HRE were stratified by the 1^st^ and 3^rd^ tertiles, respectively, from the gains in maximal power output (MPO) in response to ET and HIIT. ES: Effect size calculated as the standardized difference between HRE and LRE in standard deviation units; CI: Confidence intervals. ^LT^ Data log transformed before analysis, including ES when necessary.

^#^ Set of metabolites with correlation coefficients (*r*) between baseline values and (MPO) gains higher than 0.2.

*Difference from LRE (as determined by the 95% CI for ES that did not cross zero), bold values.

### Pathway analysis

For pathway analysis, baseline metabolites that were correlated at *r* ≥ 0.2 with MPO gains were used, separately for serum (ET: 13 metabolites; HIIT: 17 metabolites) and skeletal muscle (ET: 49 metabolites; HIIT: 13 metabolites) in each exercise program. We relied on MetaboAnalyst 3.0, ‘Homo Sapiens’ library, and used the test for Over Representation Analysis and the test for Pathway Topology Analysis to explore pathway enrichment as suggested by the profile of MPO associated metabolite concentrations.

**Fig 2:**
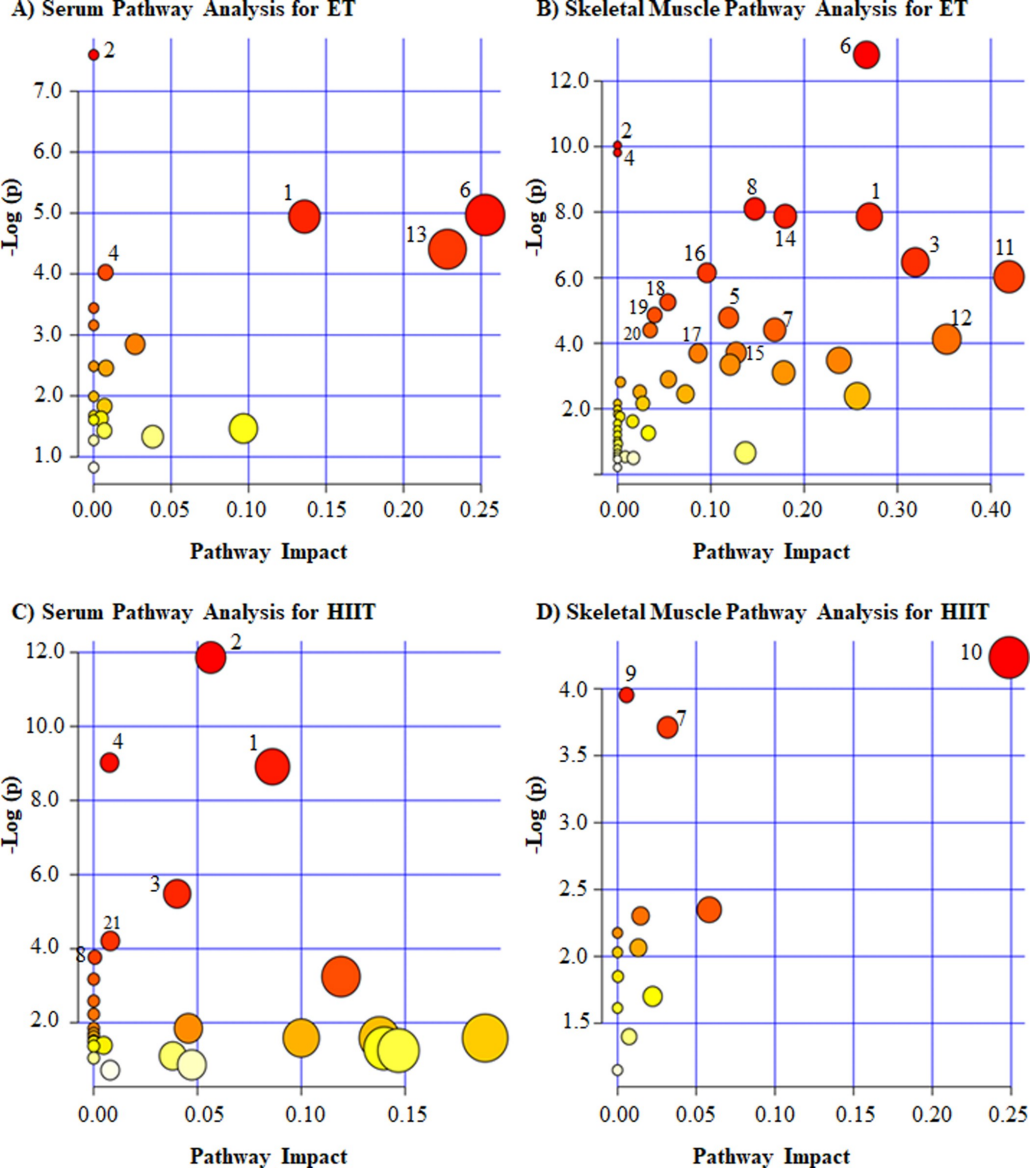
Summary of baseline serum and skeletal muscle pathways suggested to be related to MPO gains under continuous endurance training (ET) and high-intensity interval training (HIIT) programs in TIMES. The numbers in the figure panels refer to pathways that were most enriched. Pathway numbers refer to the same pathways across all 4 panels. All pathways represented in the figure had a false discovery rate of 0.1 (see text for details). The pathway impact on the horizontal axis is a score representing the relative contribution of all matched metabolites in relation to all metabolites in the given pathway. **(1)** Arginine and proline metabolism (A: glutamine, ornithine, and creatinine; B: glutamine, ornithine, proline, creatine, and creatinine; C: guanidoacetate, creatine, phosphocreatine, N-methylhydantoin, and urea); **(2)** Aminoacyl-tRNA biosynthesis (A: asparagine, glutamine, methionine and threonine; B: histidine, phenylalanine, glutamine, glycine, alanine, isoleucine, threonine, and proline; C: asparagine, histidine, phenylalanine, methionine, lysine, and tyrosine); **(3)** Glycine, serine and threonine metabolism (B: dimethylglycine, glycine, threonine, creatine, and pyruvate; C: guanidoacetate, N,N-dimethylglycine, and creatine); **(4)** Nitrogen metabolism (A: asparagine and glutamine; B: phenylalanine, glutamine, histidine, glycine, formate, and AMP; C: phenylalanine, tyrosine, asparagine, and histidine); **(5)** Phenylalanine metabolism (B: phenylalanine, pyruvate, succinate, and fumarate); **(6)** Alanine, aspartate and glutamate metabolism (A: asparagine and glutamine; B: N-acetylaspartate, alanine, pyruvate, glutamine, fumarate, and succinate); **(7)** Glyoxylate and dicarboxylate metabolism (B: isocitrate, formate, pyruvate, and succinate; D: isocitrate and glycolate); **(8)** Methane metabolism (B: glycine, formate, trimethylamine N-oxide, dimethylamine, and methylamine; C: trimethylamine and dimethylamine); **(9)** Histidine metabolism (D: anserine and τ-methylhistidine); **(10)** Glutathione metabolism (D: Glutathione and NADP+); **(11)** Pyruvate metabolism (B: pyruvate, lactate, formate, and acetate); **(12)** Glutamine and glutamate metabolism (B: glutamate and glutamine); **(13)** Glycerolipid metabolism (A: glycerol and propylene glycol); **(14)** Citrate cycle (B: succinate, isocitrate, pyruvate, and fumarate); **(15)** Purine metabolism (B: glutamine, ATP, ADP, and AMP); **(16)** Glycolysis or Gluconeogenesis (B: pyruvate, lactate, glucose, and acetate); **(17)** Propanoate metabolism (B: succinate, lactate, and β-alanine); **(18)** Taurine and hypotaurine metabolism (B: alanine, pyruvate, and acetate); **(19)** Nicotinate and nicotinamide metabolism (B: niacinamide, NAD+, pyruvate, and fumarate); **(20)** Valine, leucine and isoleucine biosynthesis (B: pyruvate, threonine, and isoleucine); **(21)** Phenylalanine, tyrosine and tryptophan biosynthesis (C: phenylalanine and tyrosine).

We observed multiple pathways significantly related to MPO gains for each exercise program (Fig 2). Here, we report on pathways that were first retained at false discovery rate threshold of 0.1 together with their “impact” score defined as the sum of connections for matched metabolites normalized by the sum of all metabolite connections within the relevant significant pathway [49]. The complete list of significant pathaways and related metabolites for each training programs are sumarized in details in Fig 2.

From the 21 observed significant pathways, five were related to MPO gains in both exercise programs (Fig 2), including metabolites supported by all the three levels of evidence, such as: arginine and proline metabolism (Fig 2A, 2B and 2C), aminoacyl-tRNA biosynthesis (Fig 2A, 2B and 2C), glycine, serine and threonine metabolism (Fig 2B and 2C), nitrogen metabolism (Fig 2A, 2B and 2C) and glyoxylate and dicarboxylate metabolism (Fig 2B and 2D).

### Summary of metabolites and pathways associated with MPO gains

The baseline metabolites most associated with MPO trainability were identified from the three levels of evidences described above: (1) correlations with gains in MPO (*r* ≥ 0.2); (2) differences between LRE and HRE and (3) contributions of the most significant pathways related to gains in MPO.

The baseline metabolites supported by all three levels of evidence were: serum glycerol (a c) (Table 9), skeletal muscle alanine (b), proline (c), threonine (d), creatinine (e), AMP (f) and pyruvate (g) for ET (Table 10). In case of HIIT, metabolites were: serum lysine (h), phenylalanine (i) and creatine (j) (Table 9), and skeletal muscle glycolate (k) (Table 10).

**Table 9:** Summary of evidence for baseline serum metabolites related to MPO gains in response to ET and HIIT in TIMES.

Serum metabolites^#^ ^$^	ET			HIIT		
	Baseline association with MPO gains	Baseline difference between LRE and HRE	Baseline pathways associated with MPO gains	Baseline association with MPO gains	Baseline difference between LRE and HRE	Baseline pathways associated with MPO gains
***Amino Acids***						
Asparagine	X		AtRNAB; Nitrogen metabolism; AAGMB	X		AtRNABs; Nitrogen metabolism
Glutamine	X		AtRNAB;			
			Nitrogen metabolism;			
			AAGMB; APM			
Histidine				X		AtRNABs;
						Nitrogen metabolism
Isoleucine						
Lysine				X	LRE < HRE	AtRNABs
Methionine	X		AtRNABs	X		AtRNAB
Phenylalanine				X	LRE > HRE	AtRNABs; PTTB
						Nitrogen metabolism;
						Phenylalanine metabolism
Threonine	X		AtRNABs			
Tyrosine				X		AtRNABs; PTTB;
						Nitrogen metabolism;
						Phenylalanine metabolism
***Carboxylic Acids***						
Creatinine	X		APM			
Guanidoacetate				X		APM; GSTM
N,N-Dimethylglycine				X		GSTM
Ornithine	X		APM			
Creatine				X	LRE < HRE	APM; GSTM
Phosphocreatine				X		APM
***Fatty Acids***						
Methylsuccinate	X	LRE < HRE				
O-Acetylcarnitine	X	LRE < HRE				
***Hydroxy Acids***						
3-Hydroxybutyrate	X	LRE < HRE	SDKB	X		SDKB
Lactate				X		
Glycolate	X					
***Imidazopyrimidines***						
Hypoxanthine	X					
***Organic Carbonic Acids***						
N-Methylhydantoin				X		APM
Urea				X		APM
***Organic Oxygen Compounds***						
Glycerol	X	LRE < HRE	Glycerolipid metabolism	X		
Trimethylamine				X		Methane metabolism
Propyleneglycol	X		Glycerolipid metabolism			
***Unclustered***						
Dimethylamine				X		Methane metabolism

ET: Continuous endurance training; HIIT: High-intensity interval training: MPO: Maximal power output; AAGM: Alanine, aspartate and glutamate metabolism biosynthesis; AtRNAB: Aminoacyl-tRNA biosynthesis: SDKB: Synthesis and degradation of ketone bodies; APM: Arginine and proline metabolism; GSTM: Glycine, serine and threonine metabolism; PTTB: Phenylalanine, tyrosine and tryptophan biosynthesis.

#Metabolites chemical taxonomy was based on class and sub-class from Human Metabolome Database.

^$^ Set of metabolites with correlation coefficients (*r*) between baseline values and changes in MPO higher than 0.2 at least one of the exercise training programs.

**Table 10:** Summary of evidence for baseline skeletal muscle metabolites related to MPO gains in response to ET and HIIT in TIMES.

Skeletal muscle^#^^$^	ET			HIIT		
	Baseline association with MPO gains	Baseline difference between LRE and HRE	Baseline pathways associated with MPO gains	Baseline association with MPO gains	Baseline difference between LRE and HRE	Baseline pathways associated with MPO gains
***Alcohols and Polyols***						
Myo-Inositol	X					
***Amino Acids***						
Alanine	X	LRE < HRE	AAGM; AtRNAB; THM			
Anserine				X		Histidine metabolism
β-Alanine	X		Propanoate metabolism			
Glutamate	X		GGM			
Glutamine	X		AAGM; AtRNAB; APM;			
			Nitrogen metabolism;			
			GGM; Purine metabolism			
Glycine	X		AtRNAB; GSTM;			
			Nitrogen metabolism;			
			Methane metabolism;			
			Glutathione metabolism			
Histidine	X		AtRNAB;			
			Nitrogen metabolism;			
			Histidine metabolism			
Isoleucine	X		AtRNAB; VLIB;			
Leucine				X		
Phenylalanine	X		AtRNAB;			
			Phenylalanine metabolism;			
			Nitrogen metabolism			
Proline	X	LRE < HRE	AAGM; AtRNAB; APM			
Threonine	X	LRE < HRE	AtRNAB; GSTM; VLIB			
***Carboxylic Acids***						
Acetate	X		Glycolysis or			
			Gluconeogenesis;			
			Pyruvate metabolism;			
			THM			
Creatine	X		APM; GSTM			
Creatinine	X	LRE < HRE	APM			
Formate	X		Nitrogen metabolism;			
			Methane metabolism;			
			Pyruvate metabolism;			
			GDM			
Fumarate	X		AAGM; Citrate cycle; NNM;			
			Butanoate metabolism;			
			Phenylalanine metabolism			
Glutathione	X		Glutathione metabolism	X		Glutathione metabolism
Isobutyrate	X	LRE > HRE				
Isocitrate	X		Citrate cycle; Glyoxylate	X		Glyoxylate and dicarboxylate metabolism
			and dicarboxylate			
			metabolism			
N,N-Dimethylglycine	X		GSTM			
N-Acetylaspartate	X		AAGM	X		
N-Acetylglutamine	X					
Ornithine	X		APM;			
			Glutathione metabolism			
Succinate	X		GDM; AAGM;			
			Citrate cycle;			
			Phenylalanine metabolism;			
			Butanoate metabolism;			
			Propanoate metabolism			
π-Methylhistidine	X		Histidine metabolism			
*τ*-Methylhistidine	X		Histidine metabolism	X		Histidine metabolism
***Fatty Acids***						
2-Hydroxyisocaproate	X					
3-Hydroxyisovalerate	X			X		
O-Acetylcarnitine	X					
***Hydroxy Acids***						
Glycolate				X	LRE < HRE	Glyoxylate and LRE > HRE
						dicarboxylate metabolism
Lactate	X		Glycolysis or			
			Gluconeogenesis;			
			Pyruvate metabolism;			
			Propanoate metabolism			
***Imidazopyrimidines***						
3-Methylxanthine	X			X		
Oxypurinol	X					
Theophylline	X					
***Nucleosides and Nucleotides***						
ADP	X		Purine metabolism			
AMP	X		Purine metabolism; LRE < HRE			
			Nitrogen metabolism			
ATP	X		Purine metabolism			
NAD+	X		NNM			
NADP+				X		Glutathione metabolism
***Organic Oxygen Compounds***						
2-Phosphoglycerate	X					
Glucose	X		Glycolysis or Gluconeogenesis			
***Organic Nitrogen Compounds***						
Carnitine	X					
Dimethylamine	X		Methane metabolism			
Histamine	X		Histidine metabolism			
Methylamine	X			X		
N-Nitrosodimethylamine	X					
Trimethylamine-N-oxide	X		Methane metabolism			
***Unclustered***						
2-Hydroxyphenylacetate	X					
Acetamide				X		
Dimethylsulfone	X			X		
Niacinamide	X		NNM			
Pyruvate	X	LRE < HRE	AAGM; Citrate cycle; GSTM; Glycolysis or Gluconeogenesis; Pyruvate metabolism; THM; Phenylalanine metabolism; GDM; VLIB; Butanoate metabolism			

ET: Continuous endurance training; HIIT: High-intensity interval training; MPO: Maximal power output; AAGM: Alanine, aspartate and glutamate metabolism; GSTM: Glycine, serine and threonine metabolism; GDM: Glyoxylate and dicarboxylate metabolism; AtRNAB: Aminoacyl-tRNA biosynthesis; GGM: Glutamine and glutamate metabolism; NNM: Nicotinate and nicotinamide metabolism; APM: Arginine and proline metabolism; THM: Taurine and hypotaurine metabolism; VLIB: Valine, leucine and isoleucine biosynthesis.

^#^Metabolites chemical taxonomy was based on class and sub-class from Human Metabolome Database.

^*$*^Set of metabolites with correlation coefficients (*r*) between baseline values and changes in MPO higher than 0.2 at least one of the exercise training programs.

The most significant pathways associated with MPO gains suggested by the metabolites for all three levels of evidence were: aminoacyl-tRNA biosynthesis (b, c, d, h, i); alanine, aspartate and glutamate metabolism (b, c, g); glycine, serine and threonine metabolism (d, g, j), arginine and proline metabolism (c, e, j); valine, leucine and isoleucine biosynthesis (d, g); taurine and hypotaurine metabolism (b, g); nitrogen metabolism (f, i), glyoxylate and dicarboxylate metabolism (g, k), phenylalanine metabolism (g, h); glycerolipid metabolism (a); phenylalanine, tyrosine and tryptophan biosynthesis (i); purine metabolism (f), pyruvate metabolism/glycolysis or gluconeogenesis/nicotinate and nicotinamide metabolism/citrate cycle (g) (S5 Table).

## Discussion

In this study, we report on baseline targeted blood and muscle metabolites in relation to MPO trainability in response to two different exercise training programs. Our key findings can be summarized as follows: (i) significant and similiar improvement in MPO with ET and HIIT programs matched for total units of exercise performed; (ii) presence of considerable inter-individual response variability to both training programs; (iii) distinct baseline serum and skeletal muscle metabolic profile associated with MPO gains within each training program as well as differences between MPO high and low responders (HRE vs LRE); (iv) specific baseline metabolites associated with MPO gains such as serum glycerol, and skeletal muscle alanine, AMP, creatinine, proline, pyruvate and threonine for ET; serum lysine, phenylalanine and creatine for HIIT as well as skeletal muscle glycolate; and (v) pathways in baseline state arguably associated with MPO trainability, involved in the metabolism of amino acids, carbohydrates and perhaps translation processes.

In TIMES, the average gains in MPO was about 21% and 24% of the baseline values after eight weeks of ET and HIIT, respectively, with the range of responses extending from 8% to 45%. Our findings agree with those of previous studies involving untrained young adults, in which MPO gains 24% (5–65%) after 6 weeks of ET [11]; and 21% after 6 weeks of HIIT [52]. Our finding of similar MPO gains after ET and HIIT programs stands in contrast to other reports which have suggested that HIIT generates higher CRF (and by extension MPO) gains compared to moderate intensity ET [18, 53]. However, the two exercise programs of the present study were perfectly matched for time, work per session, and total program units of exercise, which is not always the case in previous studies. This suggests that ET programs performed at moderate to high intensities in sedentary people (as in the present study) have the potential to induce as much improvement in cardiorespiratory fitness and exercise capacity as HIIT-based programs.

In case of ET, we observed higher baseline serum concentrations of glycerol and skeletal muscle alanine, proline, threonine and pyruvate in HRE compared to LRE. Glycerol is the backbone of triglyceride molecules, and its fasting blood levels are strongly influenced by adipose tissue lipolysis [54, 55]. High blood concentrations of glycerol during endurance exercise are thought to contribute to thermoregulatory and cardiovascular physiology, attenuating dehydration and favoring better endurance performance [56]. In TIMES, we observed consistent relationships between baseline glycerol levels and a glycerolipid metabolism pathway with MPO trainability in ET. On the other hand, we found higher baseline muscle concentrations of pyruvate in HRE. Pyruvate stands at the crossroad between cytosolic and mitochondrial metabolism and is key in the carbon flux to the citrate cycle [57, 58]. Significant associations between baseline muscle pyruvate metabolism, citrate cycle, glycolysis pathway, glyoxylate and dicarboxylate metabolism and phenylalanine metabolism with MPO trainability in ET were suggested from the metabolite profile. HRE in the ET program were also characterized by higher concentrations of alanine, threonine, proline and creatinine. Alanine, threonine and proline are blood glucose precursors as they can be converted to pyruvate or citrate cycle intermediates [59] while creatinine is partly derived from degradation of phosphocreatine, a fast source of energy for skeletal muscle contraction [60]. Interestingly, in the ET program, other pathways were suggested from the exploration of the baseline metabolite profile: glycine, serine and threonine metabolism; arginine and proline metabolism; taurine and hypotaurine metabolism; valine, leucine and isoleucine biosynthesis; and aminoacyl-tRNA biosynthesis, all seemed to be contributing to MPO trainability. All three levels of evidence also provided support for the notion that higher baseline muscle AMP (adenosine monophosphate) concentration was associated with the gains in MPO, whereas the pathway analysis revealed that baseline purine metabolism and nitrogen metabolism also associated with MPO gains. AMP is derived from ATP and ADP hydrolysis [61]. In a state of decreased energy levels (i.e., muscle contraction or fasting), AMP concentration increases activating AMP-activated protein kinase (AMPK), which stimulates pathways involved in carbohydrate and fatty acid catabolism to restore ATP levels [62, 63]. Previous studies have shown that AMPK activation is related to increased mitochondrial biogenesis and muscle adaptation to continuous endurance training [64, 65].

In the case of exposure to the HIIT program, the three lines of evidence indicated that higher concentrations of serum creatine and lower concentrations of serum lysine and phenylalanine as well as lower skeletal muscle glycolate were associated with higher MPO gains. Fasted serum creatine can be attributed mainly to liver synthesis from glycine, arginine and methionine. Skeletal muscle creatine is converted to phosphocreatine, which is a source of high-energy phosphate used to produce ATP [60]. Our pathway analysis emphasized that creatine is involved in arginine and proline metabolism and the glycine, serine and threonine metabolism which were both associated with MPO gains. Previous studies have reported that increased availability of blood and skeletal muscle creatine through supplementation can improve ventilatory threshold and submaximal power output with HIIT programs [66, 67]. Lysine is an essential amino acid converted to acetyl CoA and a precursor of carnitine, which contributes to the transportation of long-chain fatty acids into the mitochondria [68]. Phenylalanine is another essential amino acid, and its flux provides some indication of whole body protein breakdown in the postabsorptive state [69]. Previous studies have demonstrated lower plasma phenylalanine and lysine and high creatinine (derived from creatine) concentrations in healthy subjects with high CRF compared to low CRF [29]. Here, we showed that pathways in which lysine and phenylalanine participate (such as phenylalanine tyrosine and tryptophan biosynthesis, nitrogen metabolism, and aminoacyl-tRNA biosynthesis) were related to MPO trainability in HIIT. Glycolate is a hydroxy monocarboxylic acid anion that is oxidized to glyoxylate which is a precursor of oxalate [70, 71]. It has been shown that the activity of the calcium dependent ATPase which activates the transport of calcium is itself inhibited by oxalate [72, 73]. It is tempting to speculate that lower levels of glycolate may be associated with reduced inhibition of calcium dependent ATPase, perhaps contributing to CRF regulation. Furthermore, the lower skeletal muscle glycolate and its association with higher MPO gains suggested the involvement of glyoxylate and dicarboxylate metabolism pathway. Although the glycolate function in skeletal muscle is largely unknown, one recent study has shown increased glyoxylate and dicarboxylate metabolism in plantaris muscle of trained rats (5 h after last exercise training) compared to sedentary controls [74].

Our findings suggest that MPO training response to ET is influenced by baseline metabolite levels reflecting metabolism of carbohydrates, amino acids and lipids, with pyruvate playing a pivotal role. On the other hand, for HIIT, baseline and fasting levels of amino acids were associated with MPO gains, potentially reflecting a reduced protein breakdown in high MPO responders compared to low responders. Pathway analysis revealed that there were 5 pathways derived from baseline metabolite profiling that were associated with MPO gains in both exercise programs: aminoacyl-tRNA biosynthesis, arginine and proline metabolism, glycine, serine and threonine metabolism, glyoxylate and dicarboxylate metabolism, and nitrogen metabolism. These pathways are related mainly to amino acid metabolism and exhibit connectivity with aminoacyl-tRNA biosynthesis. These observations confirm the importance of both amino acid metabolism and translation processes to MPO trainability regardless of exercise program in young men. Future studies are encouraged to investigate baseline serum and skeletal muscle metabolites predicitve of CRF trainability in other populations including women and different age groups.

The present study has considerable strengths but also some limitations. Even though the study was well powered to detect the main effect of either ET or HIIT on MPO, it is based on small sample sizes for metabolomics study. Nonetheless, the current study is comparable in size to previously reported studies on an aspect or another of this topic [26, 27, 29]. An important limitation of the present research design is that it is comparing two training programs that were only 8 weeks in duration. From other research, we understand that 8 weeks of training is generally not sufficient to achieve the maximal training response for a given exercise dose [75]. Thus our findings are impacted in an unknown manner by the fact that subjects may not all have reached their maximal training response with our ET or HIIT training programs in spite of the fact that programs were matched for total unit of exercise and total work accomplished. We also speculate that because the intensity of exercise reached substantially higher peaks in the HIIT exercise sessions compared to the ET sessions, the association of baseline metabolite with the MPO training gain may be different perhaps due to specific time-course patterns of physiological adaptations in each training modality [17, 75, 76] and biological sample [77]. This would provide an explanation for the differences observed in the direction of the correlations between a number of baseline serum or muscle metabolites with MPO training response (e.g. positive correlation in one tissue but negative in the other) and observed contrast between ET and HIIT metabolic profile. A strength of the study is that it was based on the metabolite profiles in both serum and muscle. As our metabolomics exploration was targeted, a limited number of metabolites were considered. However, a major effort was made to base the metabolite and pathway analyses solely on those metabolites that exhibited excellent reproducibility. Low to moderate reproducibility of the excluded metabolites were attributed mainly to spectrum areas overlapping with other metabolites or possibly to dynamic changes in fasting metabolism and ketone body metabolism. We aimed at parsimonious solutions by focusing primarily on metabolites and pathways that were supported by all types of analytical strategies employed in this report. The discussion of our findings and conclusions are essentially based on the commonality among three lines of evidence as defined herein. Total control over diet is almost impossible to acnhieve in free-living subjects participating in exercise training studies. A strenght of the present study is that diet was controlled in the hours preceding baseline blood and muscle sampling procedures with subjects receiving a standardized evening meal and then coming to the laboratory after a 12-hour fast.

We conclude that in young sedentary men, ET programs performed at moderate to high intensities have the potential to induce as much improvement in MPO and exercise capacity as HIIT-based programs. However, the baseline serum and skeletal muscle metabolomics profile and pathways associated with MPO trainability differ between ET and HIIT programs. We observed that inter-individual response variability of MPO is associated with baseline metabolites reflecting amino acid metabolism and translation processes in both exercise programs with further associations to metabolites suggesting involvement of carbohydrate metabolism in ET program. Replication studies are warranted.

## Supporting information

S1 Checklist CONSORT checklist. (PDF)

S1 Clinical trial (PDF)

S1 Protocol Within ethics application (in Portuguese). (PDF)

S1 Translation Of relevant parts of protocol. (PDF)

S1 Changes Changes to the protocol. (PDF)

S1 Fig Heterogeneity of maximal power output (MPO) gains (absolute and relative) in response to continuous endurance training (ET) and high-intensity interval training (HIIT) in TIMES. (TIF)

S2 Fig Mean ± standard deviation of the workloads performed during all exercise sessions for both training programs in TIMES. **A)** Continuous endurance training, (n = 30; **B)** High-intensity interval training (n = 30). * Difference from 10 min in **A** or set 1 in **B** (P < 0.01).
(TIF)

S1 Table Reproducibility of serum metabolite concentrations from assays obtained from two samples drawn 15-min apart in TIMES (*n =* 11). SD: Standard Deviation; TE: Technical Error defined by the within-subject standard deviation calculated from repeated measurements; CV: Coefficient of variation derived from the technical error and the measurement mean, expressed as a percentage; ICC: Intraclass Correlation Coefficient. ^‡^ Metabolites not considered for further analysis.
(DOCX)

S2 Table Baseline serum metabolites levels for each of the three groups in TIMES. Data are mean ± standard deviation (SD) and skewness. ET: Continuous endurance training; HIIT: High-intensity interval training; CO: Control. There were no significant differences between groups. P-values from ANOVA one-way were adjusted by false discovery rate [50]. ^LT^Data log transformed before analysis.
(DOCX)

S3 Table Baseline skeletal muscle metabolites levels for each of the three groups in TIMES. Data are mean ± standard deviation (SD) and skewness. ET: Continuous endurance training; HIIT: High-intensity interval training; CO: Control. There were no significant differences between arms. P-values from ANOVA one-way were adjusted by false discovery rate (Benjamini & Hochberg, 1995). ^LT^ Data log transformed before analysis.
(DOCX)

S4 Table Baseline characteristics of participants stratified by the 1^st^ and 3^nd^ tertiles of MPO (W) gains in response to ET and HIIT in TIMES. Data are mean ± standard deviation (SD) and skewness. ET: Continuous endurance training; HIIT: High-intensity interval training; MPO: Maximal power output; HR_MAX_: Maximal heart rate; ∆: Change Pre to Post intervention. * Difference from LRE (*P* < 0.0001 by unpaired *t* Test).
(DOCX)

S5 Table Summary of serum and skeletal muscle metabolites supported by all 3 levels of evidence and related pathways associated to MPO gains in response to ET and HIIT in TIMES. MPO: Maximal power output; ET: Continuous endurance training; HIIT: High-intensity interval training.
(DOCX)
