# Supplementary material for: Association of skeletal muscle and serum metabolites with maximum power output gains in response to continuous endurance or high-intensity interval training programs: The TIMES study – A randomized controlled trial
Source: PLoS One. 2019 Feb 11;14(2):e0212115. doi: 10.1371/journal.pone.0212115 (PMC6370248; doi:10.1371/journal.pone.0212115)
Supplement: S1 Clinical trial — (PDF) [file pone.0212115.s002.pdf]

# RBR-3rh38g
# Responsivity biomarkers of Cariorespiratory Fitness to aerobic training

**Registration Date:** May 10, 2018, 10:49 a.m.
**Last Update:** June 5, 2018, 2:25 p.m.

## Study Type:

Intervention Study

## Scientific Title:

| PT-BR | EN |
|---|---|
| Biomarcadores de responsividade da Aptidão Cardiorrespiratória ao treinamento aeróbio | Responsivity biomarkers of Cariorespiratory Fitness to aerobic training |

## Trial Identification

**UTN Number:** U1111-1213-8690

**Public Title:**

| PT-BR | EN |
|---|---|
| Biomarcadores da variabilidade de resposta individual da Aptidão Cardiorrespiratória ao treinamento aeróbio | Variability biomarkers of individual responses of the Cariorespiratory Fitness to aerobic training |

**Scientific Acronym:**

**Public Acronym:**

**Secondary Identifying Numbers:**

**52997216.8.0000.5404 - CAAE**
Issuing Authority: Plataforma Brasil

**2.687.110**
Issuing Authority: Comitê De Ética Em Pesquisa Da Universidade Estadual de Campinas

## Sponsors

**Primary Sponsor:** Faculdade de Educação Física da Universidade Estadual de Campinas

**Secondary Sponsors:**

**Institution:** Laboratório Nacional de Biociências - LNBio

**Source(s) of Monetary or Material Support:**

**Institution:** Fundação de Amparo à Pesquisa do Estado de São Paulo

## Health Conditions

**Health Condition(s) or Problem(s):**

| PT-BR | EN |
|---|---|
| Estilo de Vida Sedentário | Sedentary Lifestyle |

**General Descriptors for Health Condition(s):**

| PT-BR | ES | EN |
|---|---|---|
| **C14:** Doenças cardiovasculares | **C14:** Enfermedades cardiovasculares | **C14:** Cardiovascular diseases |

**Specific Descriptors for Health Condition(s):**

| PT-BR | ES | EN |
|---|---|---|
| **F01.829.458.705:** Estilo de Vida Sedentário | **F01.829.458.705:** Estilo de Vida Sedentario | **F01.829.458.705:** Sedentary Lifestyle |

## Interventions

**Intervention Code(s)**

Other

**Interventions:**

PT-BR

Grupos Experimentais:
Treinamento intervalado de alta-intensidade (34 participantes): Oito semanas de ciclismo, 40 min/dia, 3-4 vezes/semana a 50-90% da frequência cardíaca de reserva;
Treinamento aeróbio contínuo (35 participantes): Oito semanas de ciclismo, 40 min/dia, 3-4 vezes/semana a 70-75% da frequência cardíaca de reserva;
Controle (11 participantes): Oito semanas sem a realização de qualquer programa de exercício

EN

Experimental Groups:
High-intensity interval training (35 subjects): Eight weeks of cycling, 40 min/day, 3-4 times/week at 50-90% of heart rate reserve;
Continuous endurance training (35 subjects): Eight weeks of cycling, 40 min/day, 3-4 times/week at 70-75% of heart rate reserve;
Controle (12 subjects): Eight weeks without any exercise program

**Descriptor for Intervention(s):**

| PT-BR | ES |
|---|---|
| **G11.427.410.698.277:** Exercício | **G11.427.410.698.277:** Ejercicio |

## Recruitment

**Recruitment Status:** Recruitment completed

**Recruitment Country**

Brazil

**Planned Date of First Enrollment:** 2016-04-04

**Planned Date of Last Enrollment:** 2017-04-14

| Target Sample Size: | Gender (inclusion sex): | Inclusion Minimum Age: | Inclusion Maximum Age: |
|---|---|---|---|

| | | | |
|---|---|---|---|
| 80 | M | 18 Y | 30 Y |

**Inclusion Criteria:**

PT-BR
Homens jovens sedentários saudáveis; idade entre 18 e 30 anos

EN
Young sedentary healthy men; age between 18-30 years old

**Exclusion Criteria:**

PT-BR
Fumantes; hipertensos com pressão arterial sistólica maior que 140 mm Hg e pressão arterial diastólica maior que 90 mm Hg; diabéticos com glicose de jejum maior que 7.0 mmol L-1; obesos severos definidos como índice de massa corporal maior que 33 kg m-2; dislipidêmicos baseada em medicação; com evidências de doenças do coração; desordens metabólicas; significantes condições respiratórias crônicas ou músculo esqueléticas interferindo no exercício

EN
Smokers; hypertensive with systolic blood pressure higher than 140 and diastolic blood pressure higher than 90 mm Hg; diabetic with fasting glucose higher than 7.0 mmol L-1; severe obese defined defined as body mass index higher than 33 kg m-2; dyslipidemic based on medication; com evidence of heart diseases; metabolic disorders; significant chronic respiratory conditions or musculoskeletal problems interfering with exercise

## Study Type

**Study Design:**

PT-BR
Ensaio clínico de diagnóstico, randomizado-controlado, paralelo, aberto, com três braços

EN
Diagnostic linical trial, randomized-controlled, parallel, open, with three arms.

| Expanded access program | Study Purpose | Intervention Assignment | Number of arms | Masking type | Allocation type | Study Phase |
|---|---|---|---|---|---|---|
| False | Diagnostic | Parallel | 3 | Open | Randomized-controlled | N/A |

## Outcomes

**Primary Outcomes:**

PT-BR
O desfecho primário é a mudança na aptidão cardiorrespiratória em resposta ao treinamento, mensurada pelo consumo máximo de oxigênio (ml/min) e máxima potência produzida (W) durante teste incremental até a exaustão, pré e após 8 semanas de treinamento

EN
Main outcome is the change in the cardiorespiratory fitness in response to training, which will be measured by the maximal oxygen uptake (ml/min) and maximal power output (W) performed during incremental test until exhaustion, pre and post 8-weeks training program

**Secondary Outcomes:**

PT-BR
Níveis séricos e músculo esqueléticos (mmol) do metabólitos analisados por metabolômica. Estes metabólitos serão utilizados para ajudar a entender os mecanismos associados às variações no desfecho principal (aptidão

EN
Serum and skekeletal metabolite levels (mmol) analyzed by metabolomics . These metabolites will be used to the understanding of mechanisms associated to variation on main outcome

cardiorrespiratória) em resposta aos programas de treinamento

(cardiorespiratory fitness) in response to training programs

**PT-BR**
Mudanças na composição corporal, mensurada por pletismografia para análise da massa livre de gordura e massa gorda em kilos (kg) e percentuais (%), pré e após 8 semanas de intervenção

**EN**
Changes on body composition, measured by na composição corporal, mensurada by plethysmograph for analysis of fat-free mass and fat mass in kilograms (kg) and percentages (%) pre and post 8-weeks training program

**PT-BR**
Mudanças no perfil lipídico (colesterol total, triglicerídeos, HDL, LDL, VLDL em mg/dL) e hemograma (hemoglobina e glicose em mg/dL), pré e após 8 semanas de intervenção

**EN**
Changes in lipid profile (total cholesterol, triglycerides, HDL, LDL, VLDL em mg/dL) pre and post 8-weeks training program

**PT-BR**
Mudanças na modulação autonômica cardíaca mensurada pela variabilidade da frequência cardíaca com cardiofrequencímetro (POLAR). As análises serão realizadas no domínio do tempo (iRR, RMSSD e SDNN, em ms, e pNN50 em %) e no domínio da frequência (LF, HF, VLF, LF/HF, TP, em ms2 e n.u.) pré e após 8 semanas de intervenção

**EN**
Changes in cardiac autonomic modulation measured by the heart rate variability using heart rate monitor (POLAR). Analysis will be performed on time domain (iRR, RMSSD e SDNN, in ms, and pNN50 em %) and frequency domain (LF, HF, VLF, LF/HF, TP, em ms2 e n.u.) pre and post 8-weeks training program

## Contacts


**Contacts for Public Queries**

**Full Name:** Alex Castro

**Address:** Avenida Érico Veríssimo, 701

**City:** Campinas / Brazil

**Zip Code:** 13083-851

**Telephone:** +55-019-35216625

**E-mail:** ax.castro@yahoo.com.br

**Affiliation:** Faculdade de Educação Física da Universidade Estadual de Campinas

**Full Name:** Mara Patrica Traina Chacon-Mikahil

**Address:** Av. Érico Veríssimo, 701

**City:** Campinas / Brazil

**Zip Code:** 13083-851

**Telephone:** +55-019-35216625

**E-mail:** marapatricia@fef.unicamp.br

**Affiliation:** Faculdade de Educação Física da Universidade Estadual de Campinas

**Contacts for Scientific Queries**

**Full Name:** Alex Castro

**Address:** Avenida Érico Veríssimo, 701

**City:** Campinas / Brazil

**Zip Code:** 13083-851

**Telephone:** +55-019-35216625

**E-mail:** ax.castro@yahoo.com.br

**Affiliation:** Faculdade de Educação Física da Universidade Estadual de Campinas

**Contact(s) for Site Queries**

**Full Name:** Alex Castro

**Address:** Avenida Érico Veríssimo, 701

**City:** Campinas / Brazil

**Zip Code:** 13083-851

**Telephone:** +55-019-35216625

**E-mail:** ax.castro@yahoo.com.br

**Affiliation:** Faculdade de Educação Física da Universidade Estadual de Campinas

### Additional Links:

Download as ICTRP format

Download as OpenTrials XML format

OpenTrials v1.2
