# Supplementary material for: Association of skeletal muscle and serum metabolites with maximum power output gains in response to continuous endurance or high-intensity interval training programs: The TIMES study – A randomized controlled trial
Source: PLoS One. 2019 Feb 11;14(2):e0212115. doi: 10.1371/journal.pone.0212115 (PMC6370248; doi:10.1371/journal.pone.0212115)
Supplement: S1 Protocol — (PDF) [file pone.0212115.s003.pdf]

PROTOCOLO

Título: Biomarcadores de responsividade da aptidão cardiorrespiratória ao treinamento aeróbio: uma abordagem metabolômica

Pesquisadores:

- Dra. Mara Patrícia Traina Chacon-Mikahil

- Me. Alex Castro

Protocolo aceito pelo comitê de ética da Universidade Estadual de Campinas:

- Número do processo: 2.717.688; CAAE: 52997216.8.0000.5404.

OBJETIVO GERAL

Objetivo Geral 1: Investigar a resposta metabólica à uma sessão aguda de treinamento aeróbio contínuo (TAC) e de treinamento aeróbio intervalado (TAI) em diferentes intervalos de tempo (*time course*).

Objetivo Geral 2: Investigar biomarcadores músculo esqueléticos, salivares e sanguíneos relacionados a mudanças induzidas pelo treinamento aeróbio sobre a aptidão cardiorrespiratória.

Objetivos Específicos

- Identificar a assinatura metabólica sanguínea e salivar de uma sessão aguda de TAC e TAI;

- Identificar o momento no qual há uma maior expressão do metaboloma na saliva e no sangue após uma sessão aguda de TAC e TAI;

- Avaliar o metabolismo aeróbio, anaeróbio lático e anaeróbio alático, assim como, o gasto energético total durante uma sessão aguda de TAC e TAI;

- Identificar a assinatura metabólica músculo esquelética, salivar e sanguínea representada por alterações no perfil metabólico de altos e baixos respondedores aos ganhos, induzidos pelo TAC e TAI, sobre a aptidão caridiorrespiratória;

- Identificar biomarcadores músculo esqueléticos, salivares e sanguíneos preditores da magnitude de mudança da aptidão caridiorrespiratória em resposta ao TAC e TAI;

- Analisar a associação entre biomarcadores metabólicos músculo esqueléticos, salivares e sanguíneos;

- Identificar biomarcadores de resposta aguda a uma sessão de TAC e TAI preditores das mudanças induzidas pelo treinamento aeróbio sobre a aptidão caridiorrespiratória;

- Analisar se o estatus de alto e baixo respondedor aos ganhos da aptidão caridiorrespiratória é dependente do tipo de treinamento (TAC e TAI);

- Associar a modulação autonômica às mudanças induzidas pelo treinamento sobre a aptidão caridiorrespiratória;

- Associar a expressão de metabólitos no sangue, saliva e músculo à expressão de genes e proteínas musculares relacionados à síntese e biogênese mitocondrial.

MÉTODOS

Amostra

A amostra será constituída por 85 voluntários homens, com idade entre 18 e 30 anos, recrutados a partir de divulgação feita pelo portal da Universidade na internet, além de outros meios de comunicação.

Os voluntários serão convidados a participar do presente estudo e serão informados dos procedimentos experimentais, riscos, desconfortos e benefícios da investigação, e se consentirem com a participação, assinarão um termo de consentimento livre e esclarecido (TCLE) que será analisado pelo Comitê de Ética em Pesquisa da Universidade Estadual de Campinas. Como parte da triagem, todos os voluntários serão submetidos à anamnese clínica e eletrocardiograma de repouso e de esforço máximo em esteira se necessário, administrados por médico cardiologista.

Os critérios de inclusão serão: não ter praticado atividade física regular (mais que dois dias na semana) nos últimos 3 meses; e possuir índice de massa corporal (IMC) entre 18,5-29,9 kg/m^2. Os critérios de exclusão serão: presença de desordens

cardiovasculares e osteomioarticulares, diabetes e hipertensão; fazer uso de fármacos de uso controlado ou de substâncias que causam dependência química; não ser aprovado no eletrocardiograma; não assinar o TCLE; possuir consumo máximo de oxigênio superior a 45 ml/kg/min; apresentar frequência inferior a 85 % nas sessões de treinamento e/ou ausência em mais de três sessões de treinamento consecutivas.

Desenho Experimental

Desenho Experimental para o objetivo geral 1

Para atender ao objetivo geral 1 serão recrutados 15 voluntários (*n* necessário para promover um poder (1-β) de pelo menos 80 % nas comparações a serem realizadas, considerando o desenho experimental, assumindo taxa de erro tipo I (α) de 5 % e um tamanho de efeito conservador moderado de f = 0,3).

Previamente à realização do estudo os participantes serão familiarizados com o ambiente de coleta e protocolo de teste. No dia seguinte, serão registradas as medidas antropométricas e realizada a avaliação cardiorrespiratória. Após 72 h, os participantes serão submetidos a três sessões experimentais em um *design cross-over* (Figura 1): sessão controle (CO), sessão TAC e sessão TAI. As sessões serão realizadas em ordem randomizada e separadas por intervalo de 48-72 h. A sessão de TAC será constituída de 40 min de exercício em cicloergômetro em intensidade correspondente a 75 % do VO_{2MAX} de reserva (Gormley, Swain et al. 2008, Garber, Blissmer et al. 2011). A sessão de TAI será composta por 40 min de exercício em cicloergômetro, em intensidades alternadas de 95 % e 50 % do VO_{2MAX} de reserva, sendo realizadas 5 séries de 4 min em cada intensidade. Na sessão CO os participantes serão mantidos em repouso durante 40 min sobre o cicloergômetro. Em repouso, serão coletadas amostras de sangue venoso e

saliva em diferentes momentos: pré1 (-15 min), pré2 (-0 min) e após 0 min, 15 min, 30 min, 60 min, 90 min e 180 min decorridos de cada uma das sessões TAC, TAI e CO.

Adicionalmente, para que seja mensurado o metabolismo predominante durante as sessões de treinamento, será coletada uma amostra de sangue da artéria superficial do lóbulo da orelha (25 µl), pré (-0 min) e após 3 min e 5 min decorridos do exercício, para a determinação da concentração pico de lactato no plasma sanguíneo. O consumo de oxigênio será continuamente coletado por meio de um sistema metabólico de análise de gases expirados, durante 5 min de repouso prévio ao exercício, ao longo de toda a sessão experimental (40 min), e durante 10 min de repouso após a sessão. A frequência cardíaca será monitorada continuamente por frequencímetro, juntamente à coleta de gases expirados.

No dia anterior às sessões experimentais os voluntários receberão refeição padronizada para minimizar possíveis efeitos da dieta sobre o metabolismo, assim como, serão orientados a não realizar atividades físicas vigorosas.

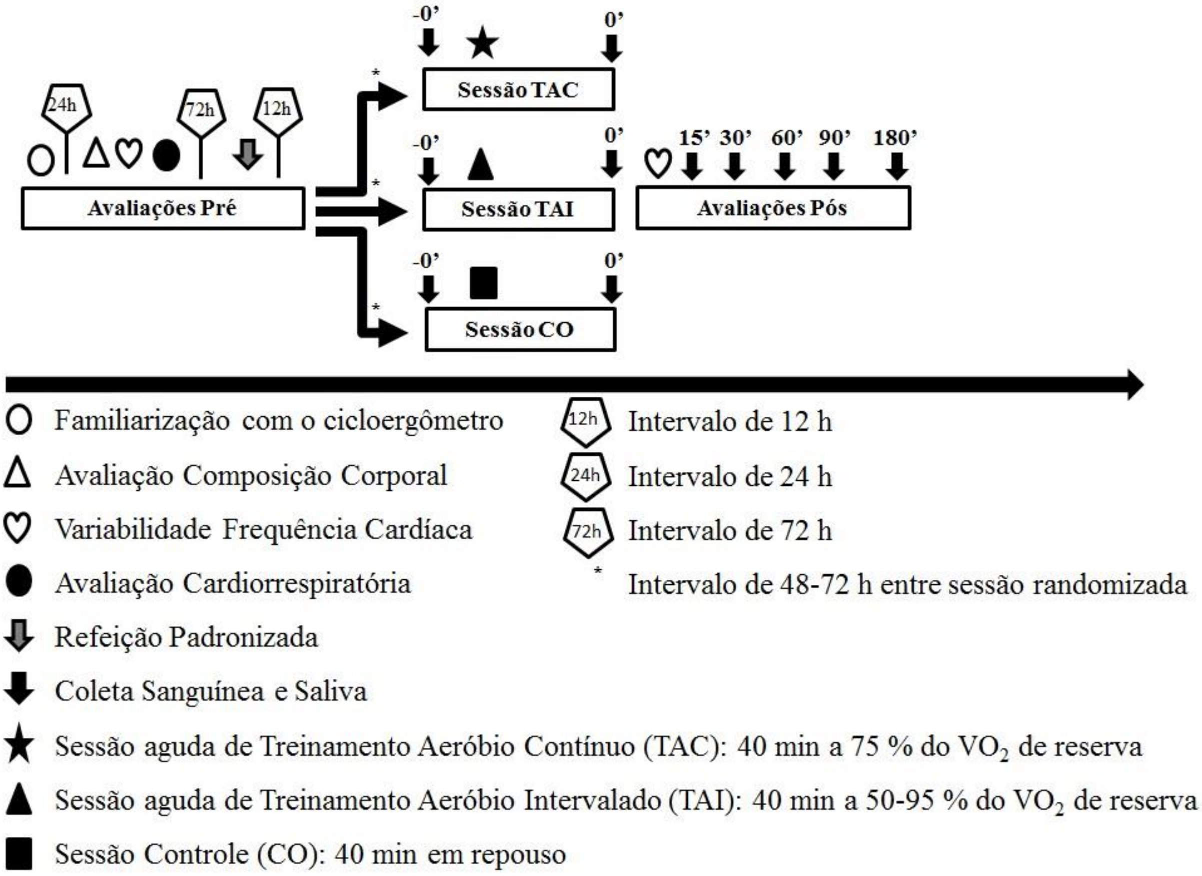

Figura 1. Desenho experimental para o objetivo geral 1.

Desenho Experimental para o objetivo geral 2

Para atender ao objetivo geral 2 serão recrutados 70 voluntários (*n* necessário para promover um poder (1-β) de pelo menos 80 % nas comparações a serem realizadas, assumindo tamanho de efeitos conservador moderados de $f = 0,3$ (para o desenho de teste F) e r = 0,5 (para desenhos correlacionais), taxa de erro do tipo I de 5 % e considerando perda amostral (*drop-out*) estimada em cerca de 15 % ao longo da intervenção.

Após a triagem, todos os voluntários serão orientados a não consumirem alimentos contendo cafeína e/ou álcool, assim como, a não realizar atividade física

vigorosa nas últimas 24 h previamente às avaliações do estudo. Adicionalmente, os voluntários serão orientados a manter seus hábitos nutricionais ao longo do estudo.

Previamente ao treinamento serão realizadas coletas de sangue, saliva e biópsia do músculo vasto lateral. Estas avaliações serão precedidas por período de 12 h de jejum após refeição padronizada. Após 72 h, será realizada avaliação da variabilidade da frequência cardíaca seguida por avaliação cardiorrespiratória. Após 48 h, será realizada a avaliação da composição corporal (pletismografia) seguida pelo re-teste da avaliação cardiorrespiratória. Após 72 h decorridas da última avaliação pré, os voluntários serão alocados aleatoriamente aos grupos: CO (n = 10) que não receberá intervenção, TAC (n = 30) e TAI (n = 30), os quais ambos receberão 8 semanas de treinamento. Pre e após (5 min e 30 mim) da primeira e última sessão de TAC e TAI, serão coletadas amostras de sangue e saliva para a identificação de biomarcadores de resposta aguda ao exercício e preditores da performance aeróbia. Na 5ª semana, após a primeira etapa de treinamento, será realizada novamente a avaliação cardiorrespiratória para o ajuste da intensidade de treinamento. Ao final, do treinamento, após 48 h da última sessão de treinamento, serão repetidas todas as avaliações referidas anteriormente no momento pré (Figura 2).

Após o treinamento, os grupos TAC e TAI serão subdivididos em quatro novos grupos a partir de análise de agrupamentos (*k-means cluster*), sendo classificados com base no grau de resposta ao VO_{2MAX} em: TAC alto respondedor, TAC baixo respondedor, TAI alto respondedor e TAI baixo respondedor (Figura 2).

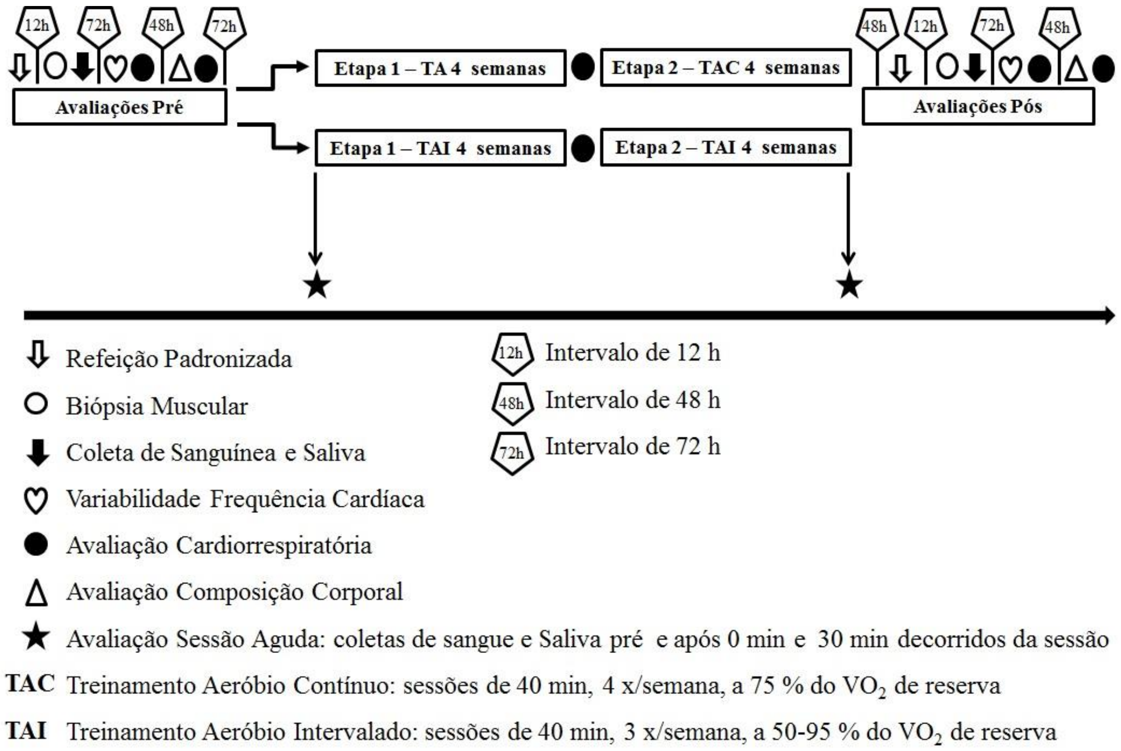

Figura 2: Desenho experimental para o objetivo geral 2.

Programa de Treinamento

O TAC e TAI terão duração de 8 semanas, sendo divido em Etapa 1 e Etapa 2, ambas com 4 semanas de duração. O TAC será composto por quatro sessões semanais de 40 min de ciclismo, em intensidade correspondente 75 % do VO_{2MAX} de reserva (Gormley, Swain et al. 2008, Garber, Blissmer et al. 2011). O TAI será composto por 3 sessões semanais de 55 min, dos quais os 5 min iniciais serão realizados a 75 % do VO_{2MAX} de reserva, seguidos por cinco séries de 5 min em cada uma das intensidades de 95 % e 50 % VO_{2max} de reserva. Ao final da Etapa 1 de ambos os treinamentos será realizada a avaliação cardiorrespiratória para o ajuste da intensidade de treinamento a ser prescrita na Etapa 2. Durante cada sessão de treinamento a intensidade será controlada por meio da FC alvo correspondente ao percentual equivalente da frequência cardíaca de reserva (FCr) (75 %) (Swain and Leutholtz 1997, Gormley, Swain et al. 2008). Esta

estratégia será utilizada devido sua vantagem em relação à prescrição baseada em percentuais relativos à FC ou VO_{2MAX}, as quais podem subestimar ou superestimar a intensidade de exercício (Garber, Blissmer et al. 2011), além de apresentar excelente correlação (r = 0,99) com o VO_2 de reserva (Swain and Leutholtz 1997) e ser efetiva na melhora da aptidão aeróbia com o treinamento (Gormley, Swain et al. 2008). Os valores de FCr e VO_2 de reserva serão calculados pela subtração dos valores em repouso dos respectivos valores máximos atingidos para cada parâmetro no teste incremental durante a avaliação cardiorrespiratória (Swain and Leutholtz 1997, Lounana, Campion et al. 2007). Todas as sessões de treinamento serão realizadas em ambiente de temperatura controlada entre 21-23 ºC.

Avaliação dos Hábitos Nutricionais

Os voluntários serão orientados a manter seus hábitos alimentares durante todo o experimento. O consumo calórico total, a quantidade e as proporções de macronutrientes (carboidratos, lipídios e proteínas) ingeridas pelos voluntários serão obtidos pré e após 4 e 8 semanas de treinamento, a partir do recordatório alimentar de três dias da semana, sendo um deles sábado ou domingo (Gerage, Forjaz et al. 2013). A quantificação dos macronutrientes será determinada pelo programa de avaliação nutricional Virtual Nutri, versão 1.0.

Coletas das Amostras Sanguíneas

As amostras de sangue venoso serão coletadas por profissional especializado, entre 7 h e 9 h da manhã, em jejum de 12 h, após refeição padronizada. Quando as coletas de sangue forem realizadas pré e após sessão aguda de treinamento aeróbio, após jejum, será fornecido aos voluntários café da manhã padronizado 2 h prévias ao momento

da coleta. Após coletadas as amostras de sangue, estas serão centrifugadas a 5000 rpm por 10 min e então as alíquotas serão armazenadas em freezer -80 °C.

Coletas das Amostras de Saliva

As amostras de saliva serão coletadas entre 7 h e 9 h da manhã, em jejum de 12 h, após refeição padronizada. Previamente à coleta da saliva os voluntários irão lavar a boca com água esterilizada (3 vezes de 30 s) e então aguardar em repouso por 5 min. Após, o voluntário irá expelir a própria saliva em tubo eppendorf até completar cerca de 750 µl. Em seguida, estas amostras serão centrifugadas (1500g) por 15 min e após, elas serão armazenadas em freezer -80 °C.

Coleta das Amostras de Tecido Muscular por Biópsia

A biópsia será realizada por médico cirurgião ortopedista experiente, sobre o músculo vasto lateral do membro inferior dominante (preferido para chutar uma bola). As biópsias serão realizadas pré e após 48 h da última sessão de treinamento, após jejum de 12 h decorridos de uma refeição padronizada consumida pelos voluntários.

Previamente, a extração do tecido, a área será tricotomizada e limpa com antisséptico. Uma pequena área, sobre a região selecionada, será anestesiada com xilocaína a 2 %, injetada subcutaneamente. Após a anestesia, uma pequena incisão, de aproximadamente (0,5 cm de comprimento), será feita até a fáscia do músculo utilizando bisturi cirúrgico. A agulha de biópsia será então introduzida no músculo numa profundidade aproximada de três centímetros para obtenção da amostra de tecido muscular, aproximadamente 100-120 mg. Esta quantificação de amostra de tecido muscular seguirá a padronização usualmente descrita na literatura, tanto para análises de

metabolômica quanto para análises de expressão gênica e proteica. Após a retirada do tecido, a incisão será fechada e coberta por bandagens.

As biópsias realizadas ao final do treinamento serão realizadas ao lado da cicatriz da primeira biópsia. Após a extração, todas as amostras serão: limpas (livre de sangue e excesso de tecido conectivo); separadas em alíquotas; e imediatamente congeladas em nitrogênio líquido e armazenadas à -80 ºC para análises posteriores.

Avaliação da Composição Corporal

A composição corporal será avaliada por pletismografia de corpo inteiro (BOD POD®; Body Composition System; Life Measurement Instruments; Concord, CA). Esta técnica determina o volume corporal através de um método de deslocamento de ar que se utiliza da relação inversa entre pressão e volume, baseado na lei de Boyle que em temperatura constante a pressão absoluta e o volume de um gás são inversamente proporcionais (McCrory, Gomez et al. 1995). A partir destes dados, com a densidade corporal serão estimados os percentuais de gordura e massa magra pela equação de Siri (Siri 1993).

Avaliação Cardiorrespiratória

A avaliação do consumo máximo de oxigênio (VO_{2MAX}) e máxima potência produzida (MPO) será realizada em cicloergômetro com frenagem eletromagnética (Corival 400, Quinton® Instrument Co., Groningen, Holanda) e a mensuração do consumo de oxigênio (VO_2) e dióxido de carbono produzido (VCO_2) serão feitas por meio de espirometria de circuito aberto, continuamente respiração a respiração, através de um sistema metabólico de análise de gases (CPX, Medical Graphics, St. Paul, Minnesota, USA).

Inicialmente serão coletadas amostras de respiração em repouso por 10 min. Em seguida, será conduzido o teste, composto por um aquecimento de 3 min, com carga inicial de 50 W em cadência de 70 rpm, seguido por incrementos de 25 W a cada min. O teste será interrompido quando o voluntário não for capaz de continuar e/ou não sustentar a cadência de 70 rpm, apesar do encorajamento verbal, ou apresentar condições de risco para a manutenção do exercício (Thompson, Arena et al. 2013).

A frequência cardíaca (FC) será monitorada continuamente por frequencímetro (Polar, Finlândia) e a percepção subjetiva de esforço registrada no 15 s de cada estágio por meio da escala de Borg (Borg and Linderho 1967).

Para a validação do teste, pelo menos dois dos seguintes critérios deverão ser atingidos: (1) Razão das trocas respiratórias (RER = CO2/O2) maior que 1.15 (MacDougall, Wenger et al. 1983); (2) um platô no VO_{2MAX}, caracterizado por um aumento ≤ 2.1 $ml.kg^{-1}$ no consumo de oxigênio (Howley, Bassett et al. 1995); (3) FC maior do que 90 % do máximo predito para a idade (Tanaka, Monahan et al. 2001); (4) escala de percepção subjetiva de esforço de Borg igual ou superior a 17 (Howley, Bassett et al. 1995).

O VO_{2MAX} será determinado a partir da maior média de 30 s dos valores de VO_2 registrados ao final do teste. A MPO será determinada como sendo o maior valor de potência produzido por unidade de tempo ao final do teste. O cálculo dos valores de VO_2 e FC de repouso serão realizados a partir da média dos valores registrados nos últimos 60 s da coleta de repouso, previamente ao teste (Swain and Leutholtz 1997).

Avaliação da Variabilidade da Frequência Cardíaca

Para a aquisição dos dados da variabilidade da frequência cardíaca, será realizado o registro da FC de repouso, entre 7 h e 12 h da manhã, em ambiente com temperatura

controlada entre 21-23 ºC. Será utilizado para o registro cardiofrequencímetro específico para obtenção dos registros batimento-a-batimento correspondentes aos intervalos entre ondas R consecutivas (iRR) (Gamelin, Berthoin et al. 2006, Nunan, Donovan et al. 2009). Antes do início do protocolo todos os procedimentos serão esclarecidos na tentativa de eliminar qualquer elemento estressor.

Para aquisição dos dados de variabilidade da frequência cardíaca o voluntário permanecerá em repouso, na posição supina, durante 15 min, respirando normalmente. A partir dos tacogramas dos iRR (intervalos entre ondas R) será realizada a análise da variabilidade da frequência cardíaca no: domínio do Tempo (DT), sendo analisado índices tais como a média iRR, SDNN e RMSSD; e domínio da Frequência (DF) no qual serão analisados índices como LF, HF, LF/HF, a partir dos espectros de frequência obtidos dos iRR, conforme procedimentos padronizados pela literatura (1996).

Preparação das Amostras de Sangue para Metabolômica

Previamente a análise, será realizada a lavagem do filtro 3kDa (Amicon Ultra) com a aplicação 500 µl de H_2O Milli-Q. Após este filtro com H_2O Milli-Q será centrifugado a 14.000 rpm, por 10 min a 4 ºC. Este processo será repetido por cinco vezes. Após a quinta lavagem, será realizado o spin (inversão do filtro e rotação de 8.000 rpm durante 5 s), com o intuito de eliminar qualquer resquício de H_2O Milli-Q. Após o spin, será adicionado ao filtro 350 µl de soro previamente armazenado, sendo centrifugado a 14.000 rpm, por 45 min a 4 ºC. Após esse período será recuperado o soro filtrado (200 µl). Posteriormente, o soro filtrado (200 ml) será adicionado ao tubo de RMN de 5 mm (Wilmad). Juntamente a esta solução, também será acrescentado um tampão fosfato (60 µl, Fosfato de Sódio Monobásico, NaH2PO4 H20-137,99g/mol; Fosfato de Sódio Dibásico, Na2HPO3- 141,96g/mol) (padronização do Ph), TSP ((ácido

3-(trimetil-silil)-2,2',3,3‘ tetradeuteropropiônico ou TMSP-d4, a 50 mmol/L em D2O (6,06µl) (referência interna) e 340 µl de H_2O Milli-Q.

Preparação das Amostras de Saliva para Metabolômica

Previamente a análise, será realizada a lavagem do filtro 3kDa (Amicon Ultra) com a aplicação 500 µl de H_2O Milli-Q. Após este filtro com H_2O Milli-Q será centrifugado a 14.000 rpm, por 10 min a 4 ºC. Este processo será repetido por cinco vezes. Após a quinta lavagem, será realizado o spin (inversão do filtro e rotação de 8.000 rpm durante 5 s), com o intuito de eliminar qualquer resquício de H_2O Milli-Q. Após o spin, será adicionado ao filtro 750 µl de saliva, sendo centrifugado a 14.000 rpm, por 45 min a 4 ºC. Após esse período será recuperada a saliva filtrada (500 µl) livre de macromoléculas e quaisquer corpúsculos. Posteriormente, a saliva filtrada (500 µl) será adicionado ao tubo de RMN de 5 mm (Wilmad). Juntamente a esta solução, também será acrescentado 50 µl de D_2O e 5 µl NaN_3 (Santone, Dinallo et al. 2014).

Preparação das Amostras de Tecido Muscular para Metabolômica

As amostras serão processadas seguindo o protocolo de Belle (Le Belle, Harris et al. 2002). Resumidamente, os fragmentos de tecido serão pesados e adicionados a uma solução fria de metanol/clorofórmio (2:1 v/v, total de 2,5 ml). Então os tecidos serão homogeneizados no gelo utilizando um homogeneizador e sonificador de tecido (VCX 500, Vibra-Cell, Sonics & Material Inc., USA) por 3 min com 10 s de intervalo entre cada min. Após uma solução fria de clorofórmio/água destilada (1:1 v/v, total de 2,5 ml) será adicionada às amostras. As amostras serão brevemente agitadas (para formar uma emulsão) e centrifugadas em 3,1 x 103 g por 20 min a 4 °C. A fase superior da mistura (contendo metanol, água e metabólitos polares) serão coletadas e totalmente secadas em

um concentrador de vácuo (miVac Duo Concentrator, Genevac, UK). A fase sólida restante será reidratada em 0,6 ml de óxido de deutério contendo tampão fosfato (0,1 M, pH 7,4) e 0,5 mM de TMSP-d4. As amostras serão adicionadas a um tubo de RMN de 5 mm para imediata aquisição.

Aquisição de Espectros e Quantificação dos Metabólitos

Os espectros serão adquiridos utilizando um espectrómetro de RMN Inova Agilent (Agilent Technologies Inc., Santa Clara, CA, EUA), operando a uma frequência de ressonância de 1H 600 MHz e temperatura constante de 298 K (25 °C). Um total de 256 decaimentos de indução livre (FID) será realizado. A fase espectral e as correções de base, assim como a identificação e quantificação dos metabólitos presentes nas amostras, serão realizadas utilizando o software Suite 7.6 Chenomx RMN (Chenomx Inc., Edmonton, AB, Canada), utilizando o sinal do TSP (concentração conhecida) como referência para a quantificação das concentrações dos outros metabólitos. Para inibir qualquer viés tendencioso, as amostras serão perfiladas aleatoriamente.

Análise da Expressão Gênica

Extração do RNA

A extração do RNA tecidual total será realizada com a utilização do reagente TRIZOL (Gibco®) segundo recomendações do fabricante. A integridade das amostras de RNA será verificada por eletroforese em gel de agarose denaturante. As concentrações e pureza serão determinadas espectrofotomicamente por leitura de absorbâncias a 260 e 280 nm. Razões A_{260}/A_{280} entre 1,8 – 2,0 serão consideradas de pureza satis fatória.

Transcrição Reversa

A síntese de cDNA será realizada utilizando 1 µg de RNA total por transcrição reversa com Superscript III, inibidor de RNase (RNase OUT) oligo dT e random primers, seguindo-se o protocolo recomendado pelo fabricante (Life Technologies). Inicialmente será realizada uma digestão com DNase, para eliminar qualquer contamin ação com DNA genômico, e, no final, uma digestão com RNase H, para degradar as fitas simples de RNA. O cDNA será diluído e utilizado para as reações de análise de expressão gênica por PCR quantitativo em tempo real.

PCR Quantitativo em Tempo Real (qRT-PCR)

Os níveis de expressão no músculo esquelético vasto lateral dos genes previamente selecionados serão determinados por qRT-PCR. As reações serão realizadas pelo método de incorporação de *Syber Green*, em aparelho ABI 7500 (Life Technologies). Os *primers* serão desenhados em *exons* diferentes para evitar amplificações de DNA com o auxilio do software Primer Express e sintetizados pela Exxtend. A Tabela 1 mostra as sequências dos *primers* utilizados nesse estudo. Para cada gene serão testa das diferentes concentrações finais de *primers* e a eficiência da amplificação ($E= 10^{(-1/slope)}-1$) será calculada utilizando-se diluições seriadas de um *pool* de cDNAs. As reações serão realizadas em duplicata, em um volume final de 12 µl, contendo 6 µl de SYBR Green PCR Master Mix (Applied Biosystems Life Technologies), 3 µl de cDNA e 3 µl de *primers.* Os valores dos genes serão normalizados utilizando -se genes constitutivos como controles internos. A análise da expressão gênica será realizada através da quantificação relativa da expressão em relação ao tecido normal que apresentou o maior valor de Delta Ct (menor nível de expressão) para cada gene. A equação $2^{-\Delta\Delta C}$ será utilizada para o cálculo da expressão relativa dos genes nas amostras avaliadas (LIVAK & SCHMITTGEN, 2001).

Tabela 1. Sequência de nucleotídeos dos *primers* que serão utilizados para as reações de PCR quantitativo em tempo real.

Gene	Senso	Antisenso
PGC-1α	ATGGAGTGACATCGAGTGTGCT	GAGTCCACCCAGAAAGCTGT
PGC-1α4	TCACACCAAACCCACAGAGA	CTGGAAGATATGGCACAT
IGF-1Ea	CAGCGCCACACCGACAT	TTGTTTCCTGCACTCCCTCTACT
HIF-1α	AAGTCTGCAACATGGAAGG	ATTTGATGGGTGAGGAATGG
P21	CAGCATGACAGATTTCTACC	CACACAAACTGAGACTAAGG
Ciclina D1	GAACAAGCTCAAGTGGAACC	CACAGAGGGCAACGAAGG
Tfam	GGAAAACCAAAAAGACCTCGTTCAGCTT	TTTTCCTGCGGTGAATCACCCTT
HSP72	ACCAAGCAGACGCAGATCTTC	GCCCTCGTACACCTGGATCA
MnSOD	CTGGACAAACCTCAGCCCTA	TGATGGCTTCCAGCAACTC
HK	CAGCAGAACAGCCTGGACGAGAGCAT	GTCAAACTCCTCTCGCCGGTGGAT
Murf-1	GGAGCCACCTTCCTCTTGACT	CTCAAAGCCCTGCTCTGTCTTC
Atrogin-1	GATGTTACCCAAGGAAAGAGCAGTAT	ACGGATGGTCAGTGCCCTT
Ciclofilina	TGGCAAGCATGTGGTCTTTGGGAAG	GGTGATCTTCTTGCTGGTCTTGCCATTC
GAPDH	GTGGTGCTAAGCGTGTTATCATC	GGCAGCACCTCTGCCATC

Análise da Expressão Proteica

Extração Proteica

Para extração de proteínas totais, será preparado um lisado tecidual utilizando-se o tampão de lise RIPA (50 mM de Tris-HCl ph 7,4, 150 mM de NaCl, 1 mM de EDTA, 1 % de NP-40, 0,25 % de Na-deoxicolato) e de inibidores de proteases (Sigma Chemical Corporation). O lisado será incubado em gelo por 15 min com agitações a cada 5 min no vortex para completa lise tecidual. Posteriormente, o lisado será centrifugado a 13000 rpm, a 4 °C, por 20 min). O sobrenadante correspondente ao extrato protéico será transferido para um novo tubo e a concentração total de proteína determinada através do método de Bradford (Pierce). Será analisada uma curva padrão com diluições seriadas d e albumina bovina nas concentrações de 0 a 2 mg/mL. Todas as amostras serão analisadas em duplicata em uma placa de 96 poços. A um volume de 5 µl das diluições de BSA e dos extratos protéicos diluídos 10 vezes serão adicionados 295 µl do Coomassie Plus – The Better Bradford Assay™ Kit (Pierce). A placa será incubada ao abrigo da luz, por 10

min, a temperatura ambiente. A leitura da placa será feita à 595 nm no leitor de placas Biotrak II Plate Reader (Amersham Biosciences). Os valores das absorbâncias da curva padrão BSA serão analisados e m um gráfico, e as concentrações dos extratos proteicos, determinadas a partir da equação da reta obtida.

Western Blot

Os extratos proteicos quantificados e aliquotados com 30 µg de proteína serão desnaturados a 95 °C por 5 min com tampão de Laemmli 4X (7,5 % 2 M Tris pH 6,8; 0,5 % SDS; 5 % azul de bromofenol; 26 % glicerol; 16,5 % de 2-mercaptoetanol), em um volume apropriado para a concentração final de 1X. Em seguida, as amostras serão separadas por eletroforese em gel de SDS-PAGE 15 % em tampão de corrida Tris-Glicina (250 mM Tris, 1,92 M Glicina, 1 % SDS). A corrida será realizada a 70Vdurante 20 min e posteriormente a 100V por aproximadamente 2 horas. Após este período, as proteínas serão transferidas para membranas de nitrocelulose (Pierce) por 50 min a 15V, utilizando-se o Trans-Blot SD Semi-dry Transfer Cell (Bio-Rad) e tampão de transferência (48 mM Tris, 39 mM Glicina, 0,00375 % SDS e 20 % de metanol). Em seguida, as membranas serão bloqueadas em solução de 5 % de leite desnatado em TBS-T (2,42 g/L Tris-Base, 8 g/L NaCl, acrescidos de 0,1 % de Tween-20 (Biomedicals), sob agitação, a 4 °C, por 16 horas. A incubação com os anticorpos primários diluídos em solução de bloqueio será feita durante 1-2 horas a temperatura ambiente ou a 4 °C, por 16 horas. Após a sequência de lavagens de quatro vezes por 5 min com TBS-T e uma vez com TBS por 10 min, as membranas serão incubadas com o anticorpo secundário apropriado associado à peroxidase por 1,5 h a temperatura ambiente. A detecção das proteínas na membrana será realizada por quimioluminescência com kit ECLTM Western Blotting Detection Reagents (Amersham). As membranas hibridizadas

anteriormente serão reutilizadas para novas incubações com outros anticorpos após a retirada do s anticorpos hibridizados com solução tampão (50 mM Tris pH 6,8, SDS 2 % e 0,1 M □-mercaptoetanol) durante 20 min a 50 °C sob leve agitação. Em seguida, as membranas serão lavadas por quatro vezes de 5 min com tampão TBS-T e uma vez de 10 min com tampão TBS , a temperatura ambiente sob agitação. Após este procedimento as membranas serão novamente bloqueadas e incubadas com o anticorpo contra β-actina (Sigma Chemical Corporation, diluição 1:400 0) como controle, seguindo o protocolo descrito anteriormente.

Densitometria

As análises semi-quantitativas da expressão protéica serão determinadas por densitometria da intensidade das bandas do Western Blot. Será utilizado a expressão da proteína β-actina como controle de massa para a normalização dos resultados. As densitometrias serão realizadas utilizando o software Image J Launcher (Image Processing and Analysis in Java, National Institutes of Health, EUA).

Análise Estatística

A confiabilidade relativa e absoluta das medidas será avaliada pelo coeficiente de correlação intra-classes e coeficiente de variação do erro típico, respectivamente (Hopkins 2000).

Para o objetivo geral 1, após checar a distribuição (Teste de Shapiro Wilk), homogeneidade de variâncias (Teste de Levene) e esfericidade dos dados (Teste Mauchly) serão realizadas análises de agrupamentos (*K-means cluster*), na qual os voluntários serão classificados em dois clusters resposta (“altos” e “baixos” respondedores) baseados na magnitude de mudança do VO_{2MAX} e MPO após o

treinamento. Este é um procedimento estatístico multivariado para identificar grupos homogêneos baseados em características comuns, no qual os clusters formados deverão ter baixa variância *intra-cluster* e diferenças significativa entre si. Esta análise tem sido indicada como modelo ideal para revelar a capacidade de resposta humana de forma imparcial (Bamman, Petrella et al. 2007). A partir disto, o grupo inicial (treinamento aeróbio) será separado em dois novos subgrupos denominados "altos" e "baixos" respondedores.

Desta forma, as diferenças nas respostas ao treinamento (agudas e crônicas) para cada variável dependente serão verificadas pelo teste ANOVA *mixed two way* (4 x 2): [Grupo (TAC alto respondedor, TAC baixo respondedor, TAI alto respondedor e TAI baixo respondedor) x Momento (pré e pós)]. Quando apropriado, serão aplicados testes *post hoc* de Sidak com os valores de *P* ajustados para múltiplos testes estatísticos ou taxa de falsa descoberta ao nível de 5 %, para minimizar as chances de erro do tipo I. Para identificar possíveis metabólitos (sanguíneos, salivares e músculo esqueléticos) relacionados à magnitude de mudança do VO_{2MAX} e MPO, os valores pré treinamento e pré primeira sessão de treinamento, assim como, os valores das mudanças (Δ %) nos metabólitos (agudos e crônicos) serão correlacionados à magnitude de mudança do VO_{2MAX} e MPO, por análise correlacional de Pearson ou Spermanm quando apropriado. Para estas análises será utilizado o software PASW statistics 18.0 (SPSS Inc., Chicago, USA), adotando-se nível de significância (α) de 5 % ($P < 0,05$). Após, os metabólitos que apresentarem altas correlações ($r > 0,7$) com as mudanças no VO_{2MAX} e MPO serão incluídos em um modelo de regressão linear múltipla (*Backward*) para identificar quanto da variância da ACR pode ser explicada pela variância de cada um destes metabólitos pré treinamento. Adicionalmente, a análise de associação dos metabólitos identificados e: expressão de gênes e proteínas relacionadas a biogênise mitocondrial; e modulação

autonômica, será realizada por análise correlacional de Pearson ou Spermanm quando apropriado.

Para a identificação dos biomarcadores preditores da responsividade ao consumo máximo de oxigênio será conduzida uma análise estatística multivariada dos dados. Desta forma, inicialmente, será verificada e heterocedasticidade dos dados e se necessária aplicada transformação logarítmica (Log10) a qual possibilitará maior simetria entre as curvas de distribuição dados, requerida para a aplicação de técnicas lineares (van den Berg, Hoefsloot et al. 2006).

Adicionalmente, devido à grande variabilidade comumente encontrada nos valores dos metabólitos identificados, estes serão pré-processados por meio da técnica *autoscaling* a qual ajustará cada valor de metabólito para uma amostra específica pela diferença entre seu respectivo valor e a média de todas as amostras deste metabólito, seguido da divisão pelo desvio padrão deste conjunto de amostras, fazendo-as comparáveis entre si. A seguir, será conduzida: uma análise por Componentes Principais (PCA) para observar possível segregação entre os grupos experimentais; e uma análise de reconhecimento de padrões por meio de Análise Discriminativa por Mínimos Quadrados Parciais (PLS-DA) para a construção de modelos reduzidos capazes de predizer a classificação dos grupos Alto e Baixo Respondedores ao TAC e TAI a partir do menor número de metabólitos possíveis. Serão incluídos neste modelo os metabólitos que apresentarem valores superiores a 1 para o índice VIP (*variable importance in projection*), a partir dos quais serão testados modelos reduzidos subsequentes até se obter um modelo com a maior capacidade preditiva e o menor número de variáveis possíveis. A robustez do modelo será avaliada por meio de testes de permutação (100 permutações) e validação cruzada.

Após a identificação dos metabólitos no modelo reduzido final resultante do PLS-DA, a capacidade preditiva individual destes metabólitos que poderá caracterizá-los como biomarcadores capazes de classificar corretamente os voluntários em Altos e Baixos Respondedores ao TAC e TAI, assim como, estabelecer valores de corte para esta classificação a partir dos valores PRE treinamento ou registrados durante uma única sessão de treinamento aeróbio, será analisada pela construção de curvas características de operação do receptor (ROC) e analisadas pela área sob estas curvas (AUC).

Para determinar a acurácia destes biomarcadores será verificada a taxa de verdadeiro positivo (sensibilidade), taxa de falso positivo e classificação da AUC (“perfeita”, AUC = 1; “excelente”, 0,9 ≤ AUC < 1; “boa” 0,8 ≤ AUC < 0,9; “razoável”, 0,7 ≤ AUC < 0,8; e “ruim”, AUC < 0,7). Serão sugeridos como biomarcadores os metabólitos que apresentarem alta sensibilidade, alta especificidade e AUC ≥ 0,7 para a classificação dos voluntários em Alto ou Baixo Respondedor ao TAC e TAI. Estas análises serão realizadas na plataforma on-line MetaboAnalyst (XIA; WISHART, 2011).

REFERÊNCIAS

(1996). "Heart rate variability. Standards of measurement, physiological interpretation, and clinical use. Task Force of the European Society of Cardiology and the North American Society of Pacing and Electrophysiology." Eur Heart J **17**(3): 354-381.

Bamman, M. M., J. K. Petrella, J. S. Kim, D. L. Mayhew and J. M. Cross (2007). "Cluster analysis tests the importance of myogenic gene expression during myofiber hypertrophy in humans." J Appl Physiol (1985) **102**(6): 2232-2239.

Borg, G. and H. Linderho (1967). "Perceived exertion and pulse rate during graded exercise in various age groups." Acta Medica Scandinavica **181**(S472): 194-206.

Gamelin, F. X., S. Berthoin and L. Bosquet (2006). "Validity of the polar S810 heart rate monitor to measure R-R intervals at rest." Med Sci Sports Exerc **38**(5): 887-893.

Garber, C. E., B. Blissmer, M. R. Deschenes, B. A. Franklin, M. J. Lamonte, I. M. Lee, D. C. Nieman, D. P. Swain and A. C. o. S. Medicine (2011). "American College of Sports Medicine position stand. Quantity and quality of exercise for developing and maintaining cardiorespiratory, musculoskeletal, and neuromotor fitness in apparently healthy adults: guidance for prescribing exercise." Med Sci Sports Exerc **43**(7): 1334-1359.

Gerage, A. M., C. L. Forjaz, M. A. Nascimento, R. S. Januário, M. D. Polito and E. S. Cyrino (2013). "Cardiovascular adaptations to resistance training in elderly postmenopausal women." Int J Sports Med **34**(9): 806-813.

Gormley, S. E., D. P. Swain, R. High, R. J. Spina, E. A. Dowling, U. S. Kotipalli and R. Gandrakota (2008). "Effect of intensity of aerobic training on VO2max." Med Sci Sports Exerc **40**(7): 1336-1343.

Hopkins, W. G. (2000). "Measures of reliability in sports medicine and science." Sports Med **30**(1): 1-15.

Howley, E. T., D. R. Bassett and H. G. Welch (1995). "Criteria for maximal oxygen uptake: review and commentary." Med Sci Sports Exerc **27**(9): 1292-1301.

Le Belle, J. E., N. G. Harris, S. R. Williams and K. K. Bhakoo (2002). "A comparison of cell and tissue extraction techniques using high-resolution 1H-NMR spectroscopy." NMR Biomed **15**(1): 37-44.

Lounana, J., F. Campion, T. D. Noakes and J. Medelli (2007). "Relationship between %HRmax, %HR reserve, %VO2max, and %VO2 reserve in elite cyclists." Med Sci Sports Exerc **39**(2): 350-357.

MacDougall, J. D. E., H. E. Wenger and H. E. Green (1983). Physiological testing of the elite athlete, Canadian Association of Sports Science.

McCrory, M. A., T. D. Gomez, E. M. Bernauer and P. A. Molé (1995). "Evaluation of a new air displacement plethysmograph for measuring human body composition." Med Sci Sports Exerc **27**(12): 1686-1691.

Nunan, D., G. Donovan, D. G. Jakovljevic, L. D. Hodges, G. R. Sandercock and D. A. Brodie (2009). "Validity and reliability of short-term heart-rate variability from the Polar S810." Med Sci Sports Exerc **41**(1): 243-250.

Santone, C., V. Dinallo, M. Paci, S. D'Ottavio, G. Barbato and S. Bernardini (2014). "Saliva metabolomics by NMR for the evaluation of sport performance." J Pharm Biomed Anal **88**: 441-446.

Siri, W. E. (1993). "Body composition from fluid spaces and density: analysis of methods. 1961." Nutrition **9**(5): 480-491; discussion 480, 492.

Swain, D. P. and B. C. Leutholtz (1997). "Heart rate reserve is equivalent to %VO2 reserve, not to %VO2max." Med Sci Sports Exerc **29**(3): 410-414.

Tanaka, H., K. D. Monahan and D. R. Seals (2001). "Age-predicted maximal heart rate revisited." J Am Coll Cardiol **37**(1): 153-156.

Thompson, P. D., R. Arena, D. Riebe, L. S. Pescatello and A. C. o. S. Medicine (2013). "ACSM's new preparticipation health screening recommendations from ACSM's guidelines for exercise testing and prescription, ninth edition." Curr Sports Med Rep **12**(4): 215-217.

van den Berg, R. A., H. C. Hoefsloot, J. A. Westerhuis, A. K. Smilde and M. J. van der Werf (2006). "Centering, scaling, and transformations: improving the biological information content of metabolomics data." BMC Genomics **7**: 142.
