# Supplementary material for: Association of skeletal muscle and serum metabolites with maximum power output gains in response to continuous endurance or high-intensity interval training programs: The TIMES study – A randomized controlled trial
Source: PLoS One. 2019 Feb 11;14(2):e0212115. doi: 10.1371/journal.pone.0212115 (PMC6370248; doi:10.1371/journal.pone.0212115)
Supplement: S1 Translation — (PDF) [file pone.0212115.s004.pdf]

PROTOCOL

Title: Responsivity biomarkers of cardiorespiratory fitness to aerobic training: a metabolomics approach

Researchers:

- Mara Patrícia Traina Chacon-Mikahil, PhD

- Alex Castro, MSc

Accepted protocol by the ethics committee of University of Campinas

Process number: 2.717.688; CAAE: 52997216.8.0000.5404.

Relevant points of the study protocol translated from Portuguese related with the present manuscript

Main Objective

(…)

General Objective 2: To investigate skeletal muscle, salivary and blood biomarkers related to induced changes by the aerobic training on cardiorespiratory fitness.

(…)

Methods

Sample

Eighty-five volunteer men, with age between 18 to 30 years old, recruited from disclosure in University web page, and others communication sources.

Volunteers will be invited to participate in the present study and they will be informed of experimental procedures, risks, discomforts and benefits of study. All participants will provide written informed consent (TCLE) before participation which will be analyzed by the Research Ethics Committee from University of Campinas. Before start study, all participants will be submitted to clinical anamneses and electrocardiogram at rest and maximal effort when appropriated, administered by cardiologist.

Inclusion criteria: sedentary men in the last 3 months previous to study; with body mass index between 18.5-29.9 kg/m^2. Exclusion criteria: to present evidence of heart diseases; musculoskeletal problems; diabetes; hipertension; use of medication or chemical substances; do not be approved in electrocardiogram exam; do not sign the TCLE; to present maximal oxygen uptake higher than 45 ml/kg/min; and frequency lower than 85 % of all training sessions and/or absence in more than three consecutive training sessions.

Experimental Design

(...)

Experimental Design to the Study 2

Seventy volunteers will be recruited to promote a power (1-β) of at least 80 % in comparisons within and between-subjects, assuming expected moderate effects of f = 0.3 e r = 0,5 for correlation designs, type I error of 5 %, and expecting a 15% dropout.

After recruitment, all volunteers will be instructed to abstain from consuming caffeine and alcohol, as well as, remain sedentary 24 h prior to the study's assessment. In addition, volunteers will be instructed to maintain their eating habits during the study.

Blood, saliva and skeletal muscle (vastus lateralis) samples will be collected before training at fasting state (12 h). After 72 h, heart rate variability assessment will be performed followed by the cardiorespiratory test. After 48 h will be assessed body composition using air displacement plethysmography followed by the re-test of cardiorespiratory test. Seventy-two hours later the last pre-training assessment, volunteers will be allocated randomly to the groups: control (CO, n = 10) without exercise, continuous endurance training (ET, n = 30) and high intensity interval training (HIIT, n = 30), which were submitted to 8-weeks training. (...). In the fifth week, cardiorespiratory test was repeated to adjust training loads. After 48 h of the last training session, all assessment performed at pre-training were repeated (Figure 2).

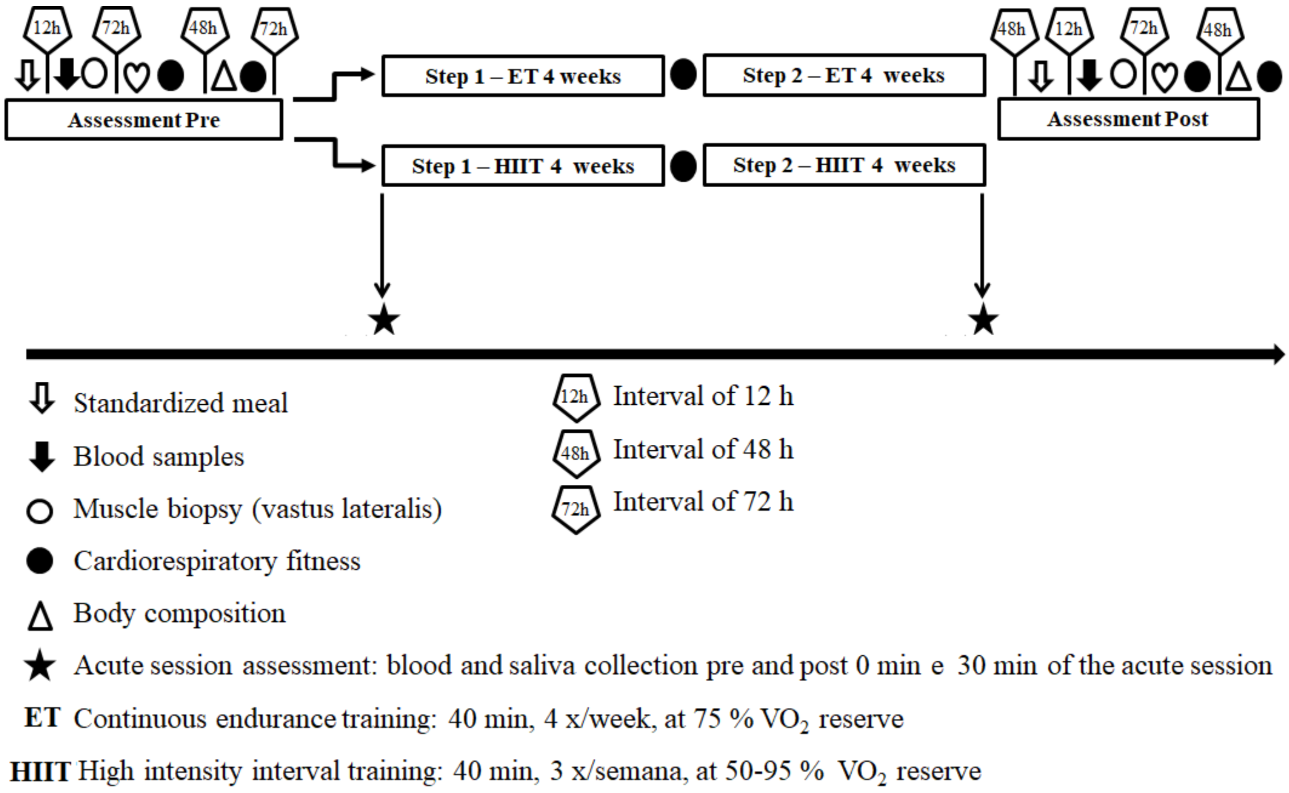

Figure 2: Experimental Design Study 2.

Training Program

The training programs were performed on cycle ergometers, 40 min per session, divided in step 1 and step 2 (4 weeks each one) for 8 weeks. For ET, participants exercised for 40 min at 75% HRR for, four times a week (Gormley *et al.*, 2008; Garber *et al.*, 2011). For HIIT, participants exercised for 40 min, at 50% HRR for 5 min, followed by 5 intervals of 5 min at 95% HRR (work phase) interspersed with 5 min at 50% HRR (recovery phase), three days a week. At the end of step 1 will be performed cardiorespiratory assessment to training intensity adjustment in step 2. During each exercise session, training intensity will be controlled using target hear rate corresponding to percentage of heart rate reserve (HRR) (Swain e Leutholtz, 1997; Gormley *et al.*, 2008). This strategy will be used because of its advantage over prescription based on HR or $VO2_{MAX}$ percentages, which may underestimate or overestimate exercise intensity (Garber *et al.*, 2011). In addition this approach present excellent correlation (r = 0,99) with VO2 reserve (Swain e Leutholtz, 1997) and is effective to improve cardiorespiratory fitness (Gormley *et al.*, 2008). HRR values will

be calculated by subtracting the values at rest from the respective maximum values reached in the incremental test during the cardiorespiratory test (Swain e Leutholtz, 1997; Lounana *et al.*, 2007). The environmental temperature will be kept around 21-23°C throughout all training sessions.

(…)

Blood Sample Collection

Venous blood samples will be collected by specialized professional, between 7:00 am and 9:00 am, in fasting state (12 h), after standardized meal. (...). After, blood samples will be centrifuged at 5000 rpm for 10 min and then aliquots stored at -80°C.

(…)

Biopsy of Skeletal Muscle Tissue

Biopsy will be collected by an orthopaedic surgeon on vastus lateralis muscle of dominant lower limb (preferred to kick a ball). Biopsies will be performed pre-training and post 48 h of last exercise training session, after standardized meal at 12 h fasting. Before tissue extraction, the skin will be trichotomized and cleansed with antiseptic. A small area over the selected region will be anesthetized with 2% xylocaine, injected subcutaneously. After anesthesia, a small incision, approximately (0.5 cm length), will be made up to the muscle fascia using surgical scalpel. The biopsy needle will then be introduced into the muscle at a depth of approximately three centimeters to obtain the muscle tissue sample, approximately 100-120 mg. This quantification of muscle tissue sample will follow the standardization usually described in the literature, both for metabolomic, gene and protein expression analysis. After tissue extraction, the incision will be closed and covered by bandages.

The biopsies performed at the end of the training will be performed next to the scar of the first biopsy. After extraction, all samples will be: clean (free of blood and excess connective tissue); separated in aliquots; and immediately frozen in liquid nitrogen and stored at -80°C for further analysis.

Body Composition Assessment

Body composition will be assessed by full body plethysmography (BOD POD®; Body Measurement Instruments; Concord, CA). This technique determines the body volume through an air displacement method that uses the inverse relationship between pressure and volume, based on Boyle's law that at constant temperature the absolute pressure and volume of a gas are inversely proportional (McCrory, Gomez et al. 1995). From these data, with the body density will be estimated the percentages of fat and lean mass by the Siri equation (Siri 1993).

Cardiorespiratory Assessment

Maximum oxygen consumption (VO_{2MAX}) and maximal power output (MPO) will be evaluated in a cycle ergometer with electromagnetic braking (Corival 400, Quinton® Instrument Co., Groningen, The Netherlands) and measurement of oxygen consumption (VO2) and dioxide of carbon produced (VCO2) will be collected by open circuit spirometry, continuously breath-to-breath through a metabolic gas analysis system (CPX, Medical Graphics, St. Paul, Minnesota, USA).

Initially breathing samples will be collected at rest for 10 min. After, the test will be started with 3 min warm-up followed by an initial load of 50 W at rate of 70 rpm, and increments of 25 W per min. The test will be discontinued when the volunteer is not able to continue and/or not maintain the cadence of 70 rpm, despite verbal encouragement, or present risk conditions for exercise maintenance (Thompson, Arena et al. 2013).

Heart rate (HR) will be continuously monitored by frequency meter (Polar, Finland) and the subjective perception of effort recorded in the last 15 s of each stage through the Borg scale (Borg and Linderho 1967)

(...)

MPO will be determined to be the highest power value produced per unit of time at the end of the test.

(...)

Blood sample preparation for metabolomics

Prior to the analysis, 3kDa filter (Amicon Ultra) will be washed with 500 µl of Milli-Q H2O. After, this filter with Milli-Q H2O will be centrifuged at 14,000 rpm, for 10 min at 4 ° C. This process will be repeated five times. After the fifth washing, the spin (filter inversion and rotation of 8.000 rpm for 5 s) will be carried out in order to eliminate any trace of Milli-Q H2O. After the spin, 350 µl of serum previously stored will be added to the filter and centrifuged at 14,000 rpm for 45 min at 4 ° C. After this time the filtered serum (200 µl) will be recovered. Subsequently, the filtered serum (200 µl) will be added to the 5 mm NMR (Wilmad) tube. This solution was diluted in a phosphate buffer (60 µl, Monobasic Sodium Phosphate, NaH2 PO4 H20-137.99 g/mol, Dibasic Sodium Phosphate, Na2HPO3-141.96 g/mol) (TS standardization), TSP (3-(trimethylsilyl)-2,2',3,3' tetradeuteropropionic acid or TMSP-d4, at 50 mmol/L in D2O (internal reference) and 340 µl of Milli-Q H2O (6.06 µl).

(...)

Muscle tissue sample preparation for metabolomics

Samples will be processed following the Belle protocol (Le Belle, Harris et al. 2002). Briefly, the tissue fragments will be weighed and added to a cold methanol/chloroform solution (2:1 v/v, total of 2.5 mL). Then the tissues will be homogenized on ice using a homogenizer and tissue sonicator (VCX 500, Vibra-Cell, Sonics & Material Inc., USA) for 3 min with 10 s interval between each min. After a cold solution of chloroform/distilled water (1: 1 v/v, total of 2.5 ml) will be added to the samples. The samples will be shaken briefly (to form an emulsion) and centrifuged at 3.1 x 103 g for 20 min at 4 ° C. The upper phase of the mixture (containing methanol, water and polar metabolites) will be collected and completely dried in a vacuum concentrator (miVac Duo Concentrator, Genevac, UK). The remaining solid phase will be rehydrated in 0.6 ml of deuterium

oxide containing phosphate buffer (0.1 M, pH 7.4) and 0.5 mM TMSP-d4. Samples will be added to a 5 mm NMR tube for immediate acquisition.

NMR Data Acquisition and Metabolite Identification

Each spectrum will be acquired using an Inova Agilent NMR spectrometer (Agilent Technologies Inc., Santa Clara, CA, USA), operating at a resonance frequency of 1 H 600 MHz and constant temperature of 298 K (25ºC). A total of 256 free induction decays (FID) will be performed. The spectral phase and base corrections, as well as the identification and quantification of the metabolites present in the samples, will be performed using the Chenomx RMN Suite software 7.6 (Chenomx Inc., Edmonton, AB, Canada), using the TSP signal as a reference for the quantification of the concentrations of the other metabolites. To inhibit any biased bias, the samples will be randomly profiled.

6. REFERÊNCIAS

Borg, G. and H. Linderho (1967). "Perceived exertion and pulse rate during graded exercise in various age groups." Acta Medica Scandinavica **181**(S472): 194-206.

Garber, C. E., B. Blissmer, M. R. Deschenes, B. A. Franklin, M. J. Lamonte, I. M. Lee, D. C. Nieman, D. P. Swain and A. C. o. S. Medicine (2011). "American College of Sports Medicine position stand. Quantity and quality of exercise for developing and maintaining cardiorespiratory, musculoskeletal, and neuromotor fitness in apparently healthy adults: guidance for prescribing exercise." Med Sci Sports Exerc **43**(7): 1334-1359.

Gormley, S. E., D. P. Swain, R. High, R. J. Spina, E. A. Dowling, U. S. Kotipalli and R. Gandrakota (2008). "Effect of intensity of aerobic training on VO2max." Med Sci Sports Exerc **40**(7): 1336-1343.

McCrory, M. A., T. D. Gomez, E. M. Bernauer and P. A. Molé (1995). "Evaluation of a new air displacement plethysmograph for measuring human body composition." Med Sci Sports Exerc **27**(12): 1686-1691.

Le Belle, J. E., N. G. Harris, S. R. Williams and K. K. Bhakoo (2002). "A comparison of cell and tissue extraction techniques using high-resolution 1H-NMR spectroscopy." NMR Biomed **15**(1): 37-44.

Lounana, J., F. Campion, T. D. Noakes and J. Medelli (2007). "Relationship between %HRmax, %HR reserve, %VO2max, and %VO2 reserve in elite cyclists." Med Sci Sports Exerc **39**(2): 350-357.

Siri, W. E. (1993). "Body composition from fluid spaces and density: analysis of methods. 1961." Nutrition **9**(5): 480-491; discussion 480, 492.

Swain, D. P. and B. C. Leutholtz (1997). "Heart rate reserve is equivalent to %VO2 reserve, not to %VO2max." Med Sci Sports Exerc **29**(3): 410-414.

Thompson, P. D., R. Arena, D. Riebe, L. S. Pescatello and A. C. o. S. Medicine (2013). "ACSM's new preparticipation health screening recommendations from ACSM's guidelines for exercise testing and prescription, ninth edition." Curr Sports Med Rep **12**(4): 215-217.
