# Supplementary material for: Association of skeletal muscle and serum metabolites with maximum power output gains in response to continuous endurance or high-intensity interval training programs: The TIMES study – A randomized controlled trial
Source: PLoS One. 2019 Feb 11;14(2):e0212115. doi: 10.1371/journal.pone.0212115 (PMC6370248; doi:10.1371/journal.pone.0212115)
Supplement: S1 Changes — (PDF) [file pone.0212115.s005.pdf]

S1 Changes. Main changes to the study protocol.

**Domain**	**Original Study Protocol**	**Implemented Change**	**Rationale/Comments**
**Training Program**	Initially, training program was divided in step 1 and step 2 (4 weeks each one) for 8 weeks. For ET, participants exercised for 40 min at 75% HRR for, four times a week (Gormley *et al.*, 2008; Garber *et al.*, 2011). For HIIT, participants exercised for 40 min, at 50% HRR for 5 min, followed by 5 intervals of 5 min at 95% HRR (work phase) interspersed with 5 min at 50% HRR (recovery phase), three days a week. At the end of step 1 will be performed cardiorespiratory assessment to training intensity adjustment in step 2.	Prior to the start of recruitment, after pilot study, we decided to promote some small changes in the training program. **Then, for ET, participants exercised at 70% HRR for 40 min, three times a week in the first four weeks (step 1); and at 75% HRR for 40 min**, 4 times a week in the last four weeks (step 2). For HIIT, participants exercised at 50% HRR for 5 min, **followed by 5 intervals of 4 min at 90% HRR (work phase) interspersed with 3 min at 50% HRR (recovery phase), three days a week**, in the first 4 weeks training (step1): **and at 60% HRR for 5 min, followed by 5 intervals of 4 min at 90% HRR and 3 min at 60% HRR**, 4 days a week in the final four weeks of training (step 2).	These small changes allowed a progressive increase in the intensity from step 1 to step 2 for both programs. In addition, we distributed the initial volume of 3 sessions of HIIT per week in 4 sessions per week, to ensure greater adherence of participants to training program.
**Blood sample preparation for metabolomics**	the filtered serum (200 µl) will be added to the 5 mm NMR (Wilmad) tube. This solution was diluted in a phosphate buffer (60 µl, Monobasic Sodium Phosphate, NaH2 PO4 H20-137.99 g/mol, Dibasic Sodium Phosphate, Na2HPO3-141.96 g/mol) (TS standardization), TSP (3-(trimethylsilyl)-2,2',3,3' tetradeuteropropionic acid	the filtered serum (**250 µl**) was added to the 5 mm NMR (Wilmad) tube. This solution was diluted in a phosphate buffer (60 µl, Monobasic Sodium Phosphate, NaH2 PO4 H20-137.99 g/mol, Dibasic Sodium Phosphate, Na2HPO3-141.96 g/mol) (TS standardization), TSP (3-(trimethylsilyl)-2,2',3,3'	We decided to increase amount of serum sample to improve the NMR spectrum resolution. We replaced Milli-Q H_2O with deuterium oxide D_2O to minimize the effects of water signal on metabolite signals in the NMR spectrum.

	or TMSP-d4, at 50 mmol/L in D2O (internal reference) and 340 µl of Milli-Q H2O (6.06 µl) was added to this solution.	tetradeuteropropionic acid or TMSP-d4, at 50 mmol/L in D2O (internal reference) and **290 µl of deuterium oxide D_2O to a 600 uL final solution.**	
**Experimental Design to the Study 2 (sample size)**	Seventy volunteers will be recruited to promote a power (1-β) of at least 80 % in comparisons within and between-subjects, assuming expected moderate effects of f = 0.3 e r = 0,5 for correlation designs, type I error of 5 %, and expecting a 15% dropout.	The sample size recruited was **80 participants**.	We decided to recruit more subjects in the study than initially planned to increase the statistical power also to small-to-moderate effects and prevent against 20% dropout.

All changes were decided before the first participant was enrolled in the study.
