# Supplementary figures and images for: Association of skeletal muscle and serum metabolites with maximum power output gains in response to continuous endurance or high-intensity interval training programs: The TIMES study – A randomized controlled trial
Source: PLoS One. 2019 Feb 11;14(2):e0212115. doi: 10.1371/journal.pone.0212115 (PMC6370248; doi:10.1371/journal.pone.0212115)

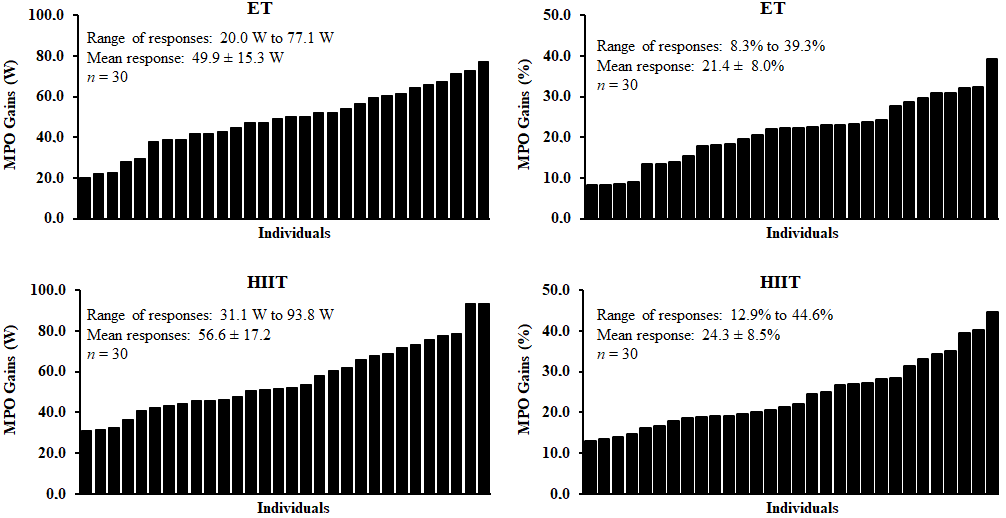

Supplement: S1 Fig — (TIF) [file pone.0212115.s006.tif]

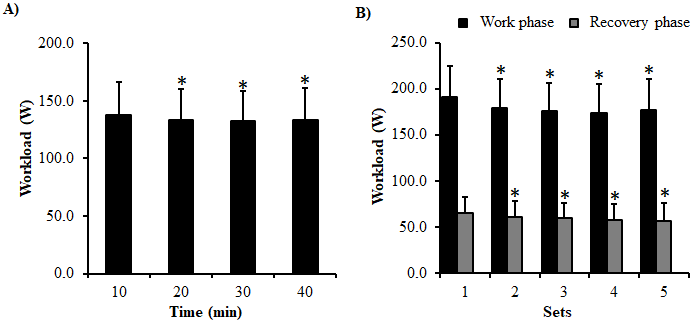

Supplement: S2 Fig — A) Continuous endurance training, (n = 30; B) High-intensity interval training (n = 30). * Difference from 10 min in A or set 1 in B (P < 0.01). (TIF) [file pone.0212115.s007.tif]
