# Supplementary material for: Association of skeletal muscle and serum metabolites with maximum power output gains in response to continuous endurance or high-intensity interval training programs: The TIMES study – A randomized controlled trial
Source: PLoS One. 2019 Feb 11;14(2):e0212115. doi: 10.1371/journal.pone.0212115 (PMC6370248; doi:10.1371/journal.pone.0212115)
Supplement: S1 Table — SD: Standard Deviation; TE: Technical Error defined by the within-subject standard deviation calculated from repeated measurements; CV: Coefficient of variation derived from the technical error and the measurement mean, expressed as a percentage; ICC: Intraclass Correlation Coefficient. ‡ Metabolites not considered for further analysis. (DOCX) [file pone.0212115.s008.docx]

| **S1 Table. Reproducibility of serum metabolite concentrations from assays obtained from two samples drawn 15-min apart in TIMES (*n =* 11).** | | | | | | |
| --- | --- | --- | --- | --- | --- | --- |
| **Serum Metabolites (mM)** | **Mean** | ± | **SD** | **TE** | **CV%** | **ICC** |
| *2-Aminobutyrate*^‡^ | 0.0096 | ± | 0.0050 | 0.0045 | 46.8 | -0.01 |
| *2-Hydroxybutyrate*^‡^ | 0.0255 | ± | 0.0086 | 0.0044 | 17.1 | 0.73 |
| 2-Hydroxyisocaproate | 0.0778 | ± | 0.0163 | 0.0046 | 5.9 | 0.95 |
| 2-Hydroxyisovalerate | 0.0104 | ± | 0.0030 | 0.0007 | 6.4 | 0.98 |
| *2-Oxoglutarate*^‡^ | 0.0330 | ± | 0.0035 | 0.0034 | 10.3 | 0.10 |
| 3-Hydroxybutyrate | 0.0357 | ± | 0.0124 | 0.0050 | 14.1 | 0.87 |
| *Acetate*^‡^ | 0.0475 | ± | 0.0299 | 0.0217 | 45.7 | 0.65 |
| *Acetoacetate*^‡^ | 0.0251 | ± | 0.0140 | 0.0071 | 28.3 | 0.79 |
| Alanine | 0.4983 | ± | 0.1075 | 0.0209 | 4.2 | 0.98 |
| Asparagine | 0.0475 | ± | 0.0114 | 0.0045 | 9.5 | 0.92 |
| Betaine | 0.0622 | ± | 0.0150 | 0.0049 | 7.9 | 0.94 |
| Carnitine | 0.0394 | ± | 0.0100 | 0.0021 | 5.5 | 0.97 |
| Choline | 0.0088 | ± | 0.0019 | 0.0010 | 11.5 | 0.85 |
| Citrate | 0.1252 | ± | 0.0280 | 0.0091 | 7.3 | 0.95 |
| Creatine | 0.0297 | ± | 0.0178 | 0.0038 | 12.9 | 0.98 |
| Creatine phosphate | 0.0025 | ± | 0.0010 | 0.0005 | 21.5 | 0.81 |
| Creatinine | 0.0912 | ± | 0.0174 | 0.0042 | 4.6 | 0.97 |
| Dimethyl sulfone | 0.0073 | ± | 0.0030 | 0.0010 | 14.3 | 0.94 |
| Dimethylamine | 0.0045 | ± | 0.0037 | 0.0002 | 4.9 | 1.00 |
| Formate | 0.0399 | ± | 0.0158 | 0.0045 | 11.4 | 0.96 |
| *Fumarate*^‡^ | 0.0015 | ± | 0.0004 | 0.0003 | 20.6 | 0.56 |
| *Glucose*^‡^ | 3.9552 | ± | 0.7297 | 0.5159 | 13.0 | 0.56 |
| Glutamine | 0.4167 | ± | 0.0717 | 0.0272 | 6.5 | 0.92 |
| Glycerol | 0.4468 | ± | 0.1678 | 0.0354 | 7.9 | 0.98 |
| Glycine | 0.2643 | ± | 0.0569 | 0.0104 | 3.9 | 0.98 |
| Glycolate | 0.0189 | ± | 0.0037 | 0.0012 | 6.1 | 0.95 |
| Guanidoacetate | 0.0743 | ± | 0.0148 | 0.0042 | 5.6 | 0.96 |
| Histidine | 0.0985 | ± | 0.0144 | 0.0043 | 4.4 | 0.95 |
| Hypoxanthine | 0.0048 | ± | 0.0017 | 0.0011 | 22.9 | 0.78 |
| Inosine | 0.0084 | ± | 0.0033 | 0.0019 | 22.9 | 0.82 |
| Isoleucine | 0.0759 | ± | 0.0187 | 0.0047 | 6.2 | 0.97 |
| Lactate | 2.2075 | ± | 0.6413 | 0.2863 | 13.0 | 0.94 |
| Lysine | 0.1307 | ± | 0.0330 | 0.0119 | 9.1 | 0.89 |
| Methionine | 0.0287 | ± | 0.0094 | 0.0059 | 20.4 | 0.79 |
| *Methylamine*^‡^ | 0.0027 | ± | 0.0005 | 0.0004 | 13.6 | 0.53 |
| Methylsuccinate | 0.0282 | ± | 0.0095 | 0.0041 | 14.6 | 0.91 |
| N,N-Dimethylglycine | 0.0039 | ± | 0.0006 | 0.0003 | 7.7 | 0.86 |
| N-Methylhydantoin | 0.0015 | ± | 0.0005 | 0.0003 | 19.4 | 0.81 |
| O-Acetylcarnitine | 0.0054 | ± | 0.0014 | 0.0004 | 7.1 | 0.95 |
| Ornithine | 0.0464 | ± | 0.0219 | 0.0098 | 21.1 | 0.84 |
| *Oxypurinol*^‡^ | 0.0837 | ± | 0.0261 | 0.0198 | 23.7 | 0.63 |
| Phenylalanine | 0.0708 | ± | 0.0087 | 0.0023 | 3.2 | 0.97 |
| Proline | 0.2670 | ± | 0.0779 | 0.0252 | 9.4 | 0.95 |
| Propylene glycol | 0.0096 | ± | 0.0030 | 0.0010 | 10.0 | 0.95 |
| Pyruvate | 0.1115 | ± | 0.0577 | 0.0211 | 18.9 | 0.94 |
| Succinate | 0.0106 | ± | 0.0039 | 0.0014 | 13.4 | 0.94 |
| Threonine | 0.1321 | ± | 0.0372 | 0.0129 | 9.7 | 0.94 |
| Trimethylamine | 0.0018 | ± | 0.0010 | 0.0002 | 10.4 | 0.98 |
| Tyrosine | 0.0892 | ± | 0.0209 | 0.0038 | 4.2 | 0.99 |
| Urea | 0.7703 | ± | 0.1848 | 0.0564 | 7.3 | 0.96 |
| Valine | 0.3103 | ± | 0.0369 | 0.0100 | 3.2 | 0.97 |
| Xanthine | 0.0242 | ± | 0.0165 | 0.0017 | 7.1 | 0.99 |
| SD: Standard Deviation; TE: Technical Error defined by the within-subject standard deviation calculated from repeated measurements; CV: Coefficient of variation derived from the technical error and the measurement mean, expressed as a percentage; ICC: Intraclass Correlation Coefficient. ^‡^ Metabolites not considered for further analysis. | | | | | | |
