# Supplementary material for: Association of skeletal muscle and serum metabolites with maximum power output gains in response to continuous endurance or high-intensity interval training programs: The TIMES study – A randomized controlled trial
Source: PLoS One. 2019 Feb 11;14(2):e0212115. doi: 10.1371/journal.pone.0212115 (PMC6370248; doi:10.1371/journal.pone.0212115)
Supplement: S2 Table — Data are mean ± standard deviation (SD) and skewness. ET: Continuous endurance training; HIIT: High-intensity interval training; CO: Control. There were no significant differences between groups. P-values from ANOVA one-way were adjusted by false discovery rate [50]. LTData log transformed before analysis. (DOCX) [file pone.0212115.s009.docx]

| **S2 Table. Baseline serum metabolites levels for each of the three groups in TIMES.** | | | | | | | | | | | | | | |
| --- | --- | --- | --- | --- | --- | --- | --- | --- | --- | --- | --- | --- | --- | --- |
| **Serum Metabolites (mM)** | **ET (*n =* 30)** | | | |  | **HIIT (*n =* 29)** | | | |  | **CO (*n =* 10)** | | | |
|  | **Mean** | **±** | **SD** | **Skewness** |  | **Mean** | **±** | **SD** | **Skewness** |  | **Mean** | **±** | **SD** | **Skewness** |
| 2-Hydroxyisocaproate | 0.067 | ± | 0.020 | 3.52 |  | 0.067 | ± | 0.018 | 0.78 |  | 0.070 | ± | 0.015 | 0.58 |
| 2-Hydroxyisovalerate | 0.010 | ± | 0.004 | 1.24 |  | 0.008 | ± | 0.003 | 0.83 |  | 0.010 | ± | 0.005 | 1.30 |
| 3-Hydroxybutyrate | 0.071 | ± | 0.077 | 2.05 |  | 0.088 | ± | 0.106 | 2.51 |  | 0.031 | ± | 0.014 | 1.27 |
| Alanine | 0.374 | ± | 0.073 | 0.89 |  | 0.351 | ± | 0.063 | 0.30 |  | 0.431 | ± | 0.106 | 1.33 |
| Asparagine | 0.038 | ± | 0.013 | -0.02 |  | 0.037 | ± | 0.010 | -0.90 |  | 0.048 | ± | 0.020 | 2.00 |
| Betaine | 0.048 | ± | 0.013 | 0.12 |  | 0.050 | ± | 0.013 | 0.76 |  | 0.055 | ± | 0.014 | 0.29 |
| Carnitine | 0.035 | ± | 0.008 | 0.41 |  | 0.034 | ± | 0.008 | 0.52 |  | 0.037 | ± | 0.012 | 2.08 |
| Choline | 0.005 | ± | 0.002 | 0.56 |  | 0.006 | ± | 0.002 | 1.33 |  | 0.004 | ± | 0.002 | 0.12 |
| Citrate | 0.099 | ± | 0.029 | 0.70 |  | 0.100 | ± | 0.029 | 2.88 |  | 0.107 | ± | 0.038 | 1.12 |
| Creatine | 0.019 | ± | 0.015 | 1.22 |  | 0.015 | ± | 0.010 | 1.29 |  | 0.020 | ± | 0.023 | 1.45 |
| Creatinephosphate | 0.005 | ± | 0.003 | 1.01 |  | 0.005 | ± | 0.003 | 0.73 |  | 0.004 | ± | 0.003 | 1.52 |
| Creatinine | 0.074 | ± | 0.019 | 1.58 |  | 0.080 | ± | 0.013 | 0.62 |  | 0.076 | ± | 0.014 | 0.73 |
| Dimethyl-sulfone | 0.004 | ± | 0.002 | 0.50 |  | 0.004 | ± | 0.003 | 1.28 |  | 0.005 | ± | 0.002 | 0.80 |
| Dimethylamine | 0.005 | ± | 0.004 | 1.68 |  | 0.005 | ± | 0.004 | 1.20 |  | 0.005 | ± | 0.004 | 1.44 |
| Formate | 0.031 | ± | 0.012 | 0.33 |  | 0.029 | ± | 0.013 | 0.44 |  | 0.046 | ± | 0.014 | 0.81 |
| Glutamine | 0.364 | ± | 0.121 | 1.98 |  | 0.346 | ± | 0.084 | -0.36 |  | 0.337 | ± | 0.073 | 1.87 |
| Glycerol | 0.202 | ± | 0.072 | 0.60 |  | 0.205 | ± | 0.069 | 0.47 |  | 0.202 | ± | 0.078 | 2.40 |
| Glycine | 0.223 | ± | 0.055 | 1.92 |  | 0.220 | ± | 0.049 | -0.08 |  | 0.243 | ± | 0.089 | 2.33 |
| Glycolate | 0.015 | ± | 0.005 | 2.19 |  | 0.015 | ± | 0.003 | 0.95 |  | 0.022 | ± | 0.009 | 1.59 |
| Guanidoacetate | 0.037 | ± | 0.017 | 0.25 |  | 0.037 | ± | 0.018 | 0.34 |  | 0.047 | ± | 0.026 | 0.18 |
| Histidine | 0.092 | ± | 0.015 | 2.77 |  | 0.090 | ± | 0.012 | 0.81 |  | 0.101 | ± | 0.025 | 2.32 |
| Hypoxanthine | 0.003 | ± | 0.001 | 0.60 |  | 0.004 | ± | 0.001 | 0.42 |  | 0.006 | ± | 0.004 | 2.18 |
| Inosine | 0.008 | ± | 0.004 | 0.76 |  | 0.009 | ± | 0.004 | 0.51 |  | 0.012 | ± | 0.009 | 1.03 |
| Isoleucine | 0.066 | ± | 0.010 | 0.65 |  | 0.066 | ± | 0.019 | 1.11 |  | 0.067 | ± | 0.017 | 0.68 |
| Lactate | 1.693 | ± | 0.395 | 0.70 |  | 1.768 | ± | 0.534 | 1.64 |  | 2.128 | ± | 0.515 | 1.34 |
| Lysine | 0.094 | ± | 0.035 | 0.76 |  | 0.093 | ± | 0.025 | 0.27 |  | 0.104 | ± | 0.038 | -1.24 |
| Methionine | 0.022 | ± | 0.006 | -0.21 |  | 0.020 | ± | 0.006 | -0.01 |  | 0.024 | ± | 0.012 | 1.19 |
| Methylsuccinate | 0.013 | ± | 0.004 | -0.72 |  | 0.015 | ± | 0.005 | 0.65 |  | 0.014 | ± | 0.004 | 0.62 |
| N,N-Dimethylglycine | 0.003 | ± | 0.001 | 0.62 |  | 0.003 | ± | 0.001 | 0.77 |  | 0.004 | ± | 0.001 | 1.31 |
| N-Methylhydantoin | 0.002 | ± | 0.001 | 0.61 |  | 0.002 | ± | 0.001 | 1.32 |  | 0.002 | ± | 0.001 | -0.01 |
| O-Acetylcarnitine | 0.005 | ± | 0.003 | 1.22 |  | 0.006 | ± | 0.003 | 1.02 |  | 0.005 | ± | 0.002 | 1.56 |
| Ornithine | 0.029 | ± | 0.013 | 1.09 |  | 0.028 | ± | 0.013 | -0.16 |  | 0.031 | ± | 0.009 | -0.48 |
| Phenylalanine | 0.061 | ± | 0.011 | 1.80 |  | 0.060 | ± | 0.011 | 1.84 |  | 0.065 | ± | 0.008 | 1.39 |
| Proline | 0.133 | ± | 0.061 | 0.15 |  | 0.133 | ± | 0.074 | 0.52 |  | 0.117 | ± | 0.061 | -0.32 |
| Propyleneglycol | 0.017 | ± | 0.004 | -0.18 |  | 0.019 | ± | 0.004 | 0.96 |  | 0.019 | ± | 0.005 | 0.63 |
| Pyruvate | 0.042 | ± | 0.019 | 0.33 |  | 0.044 | ± | 0.019 | 0.35 |  | 0.053 | ± | 0.026 | 0.72 |
| Succinate | 0.008 | ± | 0.004 | 2.68 |  | 0.009 | ± | 0.003 | 0.79 |  | 0.008 | ± | 0.004 | 1.66 |
| Threonine | 0.128 | ± | 0.033 | 2.15 |  | 0.118 | ± | 0.030 | 0.71 |  | 0.130 | ± | 0.040 | 1.16 |
| Trimethylamine | 0.002 | ± | 0.001 | 0.57 |  | 0.002 | ± | 0.001 | 0.67 |  | 0.002 | ± | 0.001 | 2.17 |
| Tyrosine | 0.074 | ± | 0.014 | 1.14 |  | 0.071 | ± | 0.013 | 0.07 |  | 0.091 | ± | 0.026 | 1.20 |
| Urea | 0.559 | ± | 0.180 | 1.74 |  | 0.579 | ± | 0.206 | 1.88 |  | 0.498 | ± | 0.168 | 0.25 |
| Valine | 0.281 | ± | 0.062 | 3.28 |  | 0.282 | ± | 0.050 | 0.31 |  | 0.297 | ± | 0.071 | 2.10 |
| Xanthine | 0.021 | ± | 0.008 | 1.10 |  | 0.025 | ± | 0.006 | 0.78 |  | 0.023 | ± | 0.013 | 0.50 |
| Data are mean ± standard deviation (SD) and skewness. ET: Continuous endurance training; HIIT: High-intensity interval training; CO: Control. There were no significant differences between groups. P-values from ANOVA one-way were adjusted by false discovery rate of 1% (Benjamini & Hochberg, 1995). ^LT^Data log transformed before analysis. | | | | | | | | | | | | | | |
