# Supplementary material for: Association of skeletal muscle and serum metabolites with maximum power output gains in response to continuous endurance or high-intensity interval training programs: The TIMES study – A randomized controlled trial
Source: PLoS One. 2019 Feb 11;14(2):e0212115. doi: 10.1371/journal.pone.0212115 (PMC6370248; doi:10.1371/journal.pone.0212115)
Supplement: S3 Table — Data are mean ± standard deviation (SD) and skewness. ET: Continuous endurance training; HIIT: High-intensity interval training; CO: Control. There were no significant differences between arms. P-values from ANOVA one-way were adjusted by false discovery rate (Benjamini & Hochberg, 1995). LT Data log transformed before analysis. (DOCX) [file pone.0212115.s010.docx]

|  |
| --- |

| **S3 Table. Baseline skeletal muscle metabolites levels for each of the three groups in TIMES.** | | | | | | | | | | | | | | |  |
| --- | --- | --- | --- | --- | --- | --- | --- | --- | --- | --- | --- | --- | --- | --- | --- |
| **Skeletal Muscle Metabolites (mM g^-1^)** | **ET (*n =* 29)** | | | |  | **HIIT (*n =* 28)** | | | |  | **CO (*n =* 10)** | | | | |
|  | **Mean** | ± | **SD** | **Skewness** |  | **Mean** | ± | **SD** | **Skewness** |  | **Mean** | ± | **SD** | **Skewness** | |
| 2-Hydroxyisocaproate ^LT^ | 0.085 | ± | 0.147 | 3.86 |  | 0.051 | ± | 0.039 | 1.80 |  | 0.049 | ± | 0.026 | 0.65 | |
| 2-Hydroxyphenylacetate | 0.062 | ± | 0.044 | 1.85 |  | 0.064 | ± | 0.035 | 1.02 |  | 0.062 | ± | 0.021 | -0.57 | |
| 2-Phosphoglycerate | 2.217 | ± | 2.382 | 2.55 |  | 2.017 | ± | 1.428 | 0.50 |  | 0.869 | ± | 0.517 | 0.73 | |
| 3-Hydroxyisovalerate ^LT^ | 0.157 | ± | 0.319 | 5.23 |  | 0.102 | ± | 0.046 | 1.94 |  | 0.094 | ± | 0.026 | -1.08 | |
| 3-Methylxanthine | 0.098 | ± | 0.063 | 1.03 |  | 0.070 | ± | 0.038 | 0.61 |  | 0.080 | ± | 0.038 | -0.15 | |
| ADP | 0.023 | ± | 0.019 | 1.98 |  | 0.020 | ± | 0.009 | 1.47 |  | 0.018 | ± | 0.009 | 1.97 | |
| AMP | 0.084 | ± | 0.058 | 1.46 |  | 0.132 | ± | 0.139 | 2.23 |  | 0.067 | ± | 0.028 | 0.34 | |
| ATP ^LT^ | 0.062 | ± | 0.071 | 4.73 |  | 0.050 | ± | 0.019 | 0.63 |  | 0.048 | ± | 0.021 | 1.01 | |
| Acetamide | 0.050 | ± | 0.023 | 0.46 |  | 0.045 | ± | 0.020 | 0.21 |  | 0.044 | ± | 0.015 | 0.47 | |
| Acetate | 0.434 | ± | 0.304 | 2.48 |  | 0.456 | ± | 0.220 | 0.71 |  | 0.369 | ± | 0.107 | -0.31 | |
| Alanine ^LT^ | 2.161 | ± | 1.406 | 3.85 |  | 1.985 | ± | 0.527 | 0.19 |  | 1.828 | ± | 0.472 | 0.99 | |
| Anserine ^LT^ | 0.115 | ± | 0.148 | 3.42 |  | 0.078 | ± | 0.068 | 2.22 |  | 0.115 | ± | 0.047 | 1.19 | |
| Betaine | 0.086 | ± | 0.086 | 1.85 |  | 0.090 | ± | 0.086 | 0.94 |  | 0.047 | ± | 0.041 | 1.33 | |
| Carnitine | 1.713 | ± | 1.076 | 0.57 |  | 1.941 | ± | 1.342 | 0.88 |  | 1.741 | ± | 1.305 | 1.13 | |
| Carnosine | 5.218 | ± | 2.118 | 1.34 |  | 5.081 | ± | 2.063 | 0.69 |  | 5.698 | ± | 1.986 | 0.27 | |
| Choline | 0.064 | ± | 0.043 | -0.09 |  | 0.075 | ± | 0.047 | 0.74 |  | 0.088 | ± | 0.041 | 0.33 | |
| Citrate | 0.223 | ± | 0.173 | 2.89 |  | 0.200 | ± | 0.116 | 0.77 |  | 0.163 | ± | 0.103 | 0.25 | |
| Creatine ^LT^ | 17.22 | ± | 13.79 | 4.31 |  | 16.14 | ± | 4.082 | 0.84 |  | 13.90 | ± | 2.400 | 1.26 | |
| Creatinephosphate ^LT^ | 15.03 | ± | 11.44 | 3.96 |  | 14.11 | ± | 5.340 | 0.51 |  | 14.36 | ± | 3.759 | -0.16 | |
| Creatinine | 0.163 | ± | 0.112 | 2.10 |  | 0.160 | ± | 0.072 | 1.66 |  | 0.162 | ± | 0.077 | 1.54 | |
| Dimethylsulfone | 0.025 | ± | 0.017 | 2.03 |  | 0.019 | ± | 0.010 | 0.26 |  | 0.026 | ± | 0.015 | 0.20 | |
| Dimethylamine ^LT^ | 0.032 | ± | 0.048 | 4.99 |  | 0.025 | ± | 0.017 | 0.65 |  | 0.031 | ± | 0.028 | 2.63 | |
| Ethyleneglycol | 0.235 | ± | 0.112 | 0.43 |  | 0.280 | ± | 0.154 | 0.89 |  | 0.290 | ± | 0.165 | 1.21 | |
| Formate ^LT^ | 0.960 | ± | 0.905 | 3.52 |  | 0.944 | ± | 0.705 | 2.76 |  | 0.707 | ± | 0.331 | 0.46 | |
| Fumarate | 0.049 | ± | 0.029 | 2.22 |  | 0.054 | ± | 0.021 | 1.06 |  | 0.038 | ± | 0.013 | 1.01 | |
| Glucose ^LT^ | 0.710 | ± | 0.480 | 3.42 |  | 0.561 | ± | 0.253 | 0.46 |  | 0.681 | ± | 0.211 | 0.19 | |
| Glutamate ^LT^ | 0.879 | ± | 1.123 | 4.88 |  | 0.802 | ± | 0.323 | 0.30 |  | 0.793 | ± | 0.238 | 0.50 | |
| Glutamine ^LT^ | 8.691 | ± | 7.111 | 4.56 |  | 8.143 | ± | 2.767 | 0.28 |  | 6.193 | ± | 1.663 | 1.00 | |
| Glutathione ^LT^ | 0.141 | ± | 0.150 | 3.91 |  | 0.146 | ± | 0.080 | 0.62 |  | 0.196 | ± | 0.106 | 0.84 | |
| Glycerol | 0.386 | ± | 0.548 | 2.75 |  | 0.361 | ± | 0.393 | 1.49 |  | 0.282 | ± | 0.236 | 1.28 | |
| Glycine ^LT^ | 0.801 | ± | 0.691 | 4.14 |  | 0.759 | ± | 0.370 | 1.65 |  | 0.578 | ± | 0.312 | 0.40 | |
| Glycolate | 0.995 | ± | 0.888 | 1.56 |  | 0.667 | ± | 0.482 | 0.43 |  | 1.097 | ± | 0.419 | 0.29 | |
| Histamine ^LT^ | 0.202 | ± | 0.324 | 4.43 |  | 0.197 | ± | 0.133 | 0.41 |  | 0.137 | ± | 0.083 | 0.11 | |
| Histidine ^LT^ | 0.333 | ± | 0.372 | 3.21 |  | 0.279 | ± | 0.121 | 0.52 |  | 0.234 | ± | 0.060 | 0.76 | |
| Isobutyrate ^LT^ | 0.100 | ± | 0.181 | 3.38 |  | 0.071 | ± | 0.064 | 1.63 |  | 0.110 | ± | 0.104 | 1.62 | |
| Isocitrate | 0.279 | ± | 0.250 | 1.91 |  | 0.217 | ± | 0.131 | 1.25 |  | 0.131 | ± | 0.066 | 0.83 | |
| Isoleucine | 0.188 | ± | 0.234 | 2.23 |  | 0.117 | ± | 0.081 | 1.73 |  | 0.130 | ± | 0.110 | 1.63 | |
| Lactate | 4.105 | ± | 2.077 | 2.52 |  | 4.526 | ± | 1.810 | 0.51 |  | 3.241 | ± | 0.994 | 1.03 | |
| Leucine ^LT^ | 0.216 | ± | 0.300 | 3.16 |  | 0.155 | ± | 0.106 | 1.07 |  | 0.159 | ± | 0.136 | 1.63 | |
| Maleate | 0.017 | ± | 0.011 | 1.50 |  | 0.015 | ± | 0.010 | 0.23 |  | 0.020 | ± | 0.008 | -0.52 | |
| Malonate ^LT^ | 0.685 | ± | 0.565 | 4.34 |  | 0.687 | ± | 0.203 | 0.54 |  | 0.604 | ± | 0.152 | 0.10 | |
| Methylamine ^LT^ | 0.063 | ± | 0.091 | 4.46 |  | 0.064 | ± | 0.041 | 1.79 |  | 0.052 | ± | 0.047 | 2.12 | |
| N,N-Dimethylglycine | 0.035 | ± | 0.023 | 2.22 |  | 0.033 | ± | 0.013 | 0.96 |  | 0.027 | ± | 0.009 | 0.34 | |
| N-Acetylaspartate | 0.063 | ± | 0.021 | 0.23 |  | 0.057 | ± | 0.016 | 0.52 |  | 0.059 | ± | 0.015 | -0.08 | |
| N-Acetylglutamine | 0.051 | ± | 0.019 | 0.14 |  | 0.053 | ± | 0.015 | 0.79 |  | 0.054 | ± | 0.012 | 1.22 | |
| N-Nitrosodimethylamine | 0.066 | ± | 0.033 | 2.64 |  | 0.072 | ± | 0.035 | 0.83 |  | 0.051 | ± | 0.013 | 0.84 | |
| NAD+ ^LT^ | 0.150 | ± | 0.110 | 3.09 |  | 0.140 | ± | 0.061 | -0.05 |  | 0.165 | ± | 0.063 | -0.14 | |
| NADP+ ^LT^ | 0.020 | ± | 0.024 | 4.54 |  | 0.018 | ± | 0.010 | 0.83 |  | 0.023 | ± | 0.010 | 0.27 | |
| Niacinamide | 0.134 | ± | 0.124 | 2.98 |  | 0.147 | ± | 0.105 | 1.22 |  | 0.064 | ± | 0.033 | -0.02 | |
| Nicotinurate | 0.043 | ± | 0.029 | 2.36 |  | 0.039 | ± | 0.020 | 2.19 |  | 0.044 | ± | 0.018 | 0.33 | |
| O-Acetylcarnitine ^LT^ | 0.577 | ± | 0.749 | 4.47 |  | 0.507 | ± | 0.296 | 1.07 |  | 0.510 | ± | 0.291 | 1.54 | |
| Ornithine ^LT^ | 0.148 | ± | 0.131 | 3.46 |  | 0.125 | ± | 0.046 | 0.67 |  | 0.108 | ± | 0.040 | -0.57 | |
| Oxypurinol ^LT^ | 0.963 | ± | 1.171 | 4.14 |  | 0.702 | ± | 0.538 | 0.78 |  | 1.129 | ± | 0.326 | 0.43 | |
| Phenylalanine | 0.104 | ± | 0.081 | 2.78 |  | 0.082 | ± | 0.022 | 0.95 |  | 0.076 | ± | 0.034 | 1.01 | |
| Proline ^LT^ | 0.623 | ± | 1.290 | 5.14 |  | 0.380 | ± | 0.190 | 0.37 |  | 0.289 | ± | 0.118 | -0.53 | |
| Pyrimidine ^LT^ | 0.027 | ± | 0.024 | 3.87 |  | 0.023 | ± | 0.010 | -0.26 |  | 0.029 | ± | 0.018 | -0.42 | |
| Pyruvate | 0.182 | ± | 0.145 | 1.43 |  | 0.229 | ± | 0.210 | 1.68 |  | 0.076 | ± | 0.027 | -0.33 | |
| Succinate ^LT^ | 0.094 | ± | 0.059 | 4.60 |  | 0.092 | ± | 0.024 | 0.47 |  | 0.089 | ± | 0.023 | 0.68 | |
| Tartrate | 0.026 | ± | 0.017 | 1.11 |  | 0.030 | ± | 0.024 | 2.52 |  | 0.046 | ± | 0.037 | 2.28 | |
| Taurine ^LT^ | 6.415 | ± | 10.90 | 5.10 |  | 5.094 | ± | 1.909 | -0.15 |  | 5.687 | ± | 1.571 | 0.42 | |
| Theophylline | 0.218 | ± | 0.163 | 1.91 |  | 0.184 | ± | 0.070 | 0.55 |  | 0.202 | ± | 0.100 | -0.17 | |
| Threonine ^LT^ | 0.311 | ± | 0.324 | 4.56 |  | 0.264 | ± | 0.103 | 0.76 |  | 0.237 | ± | 0.077 | 0.08 | |
| Trimethylamine ^LT^ | 0.039 | ± | 0.063 | 4.68 |  | 0.035 | ± | 0.022 | 1.09 |  | 0.026 | ± | 0.020 | 1.52 | |
| Trimethylamine-N-oxide^LT^ | 0.176 | ± | 0.223 | 3.83 |  | 0.155 | ± | 0.103 | 0.73 |  | 0.120 | ± | 0.065 | 0.72 | |
| Tyrosine ^LT^ | 0.064 | ± | 0.079 | 3.88 |  | 0.054 | ± | 0.032 | 0.35 |  | 0.051 | ± | 0.034 | 0.48 | |
| Valine | 0.306 | ± | 0.276 | 2.78 |  | 0.251 | ± | 0.177 | 1.22 |  | 0.152 | ± | 0.105 | -0.05 | |
| myo-Inositol | 0.657 | ± | 0.536 | 2.03 |  | 0.676 | ± | 0.436 | 1.33 |  | 0.901 | ± | 0.562 | 0.90 | |
| β-Alanine ^LT^ | 0.174 | ± | 0.263 | 4.97 |  | 0.145 | ± | 0.071 | 0.63 |  | 0.174 | ± | 0.094 | 0.64 | |
| π-Methylhistidine ^LT^ | 0.121 | ± | 0.154 | 3.17 |  | 0.087 | ± | 0.052 | 1.16 |  | 0.088 | ± | 0.031 | -0.35 | |
| *τ*-Methylhistidine | 0.364 | ± | 0.363 | 1.08 |  | 0.555 | ± | 0.709 | 3.52 |  | 0.314 | ± | 0.261 | 0.90 | |
| Data are mean ± standard deviation (SD) and skewness. ET: Continuous endurance training; HIIT: High-intensity interval training; CO: Control. There were no significant differences between arms. P-values from ANOVA one-way were adjusted by false discovery rate of 1% (Benjamini & Hochberg, 1995). ^LT^ Data log transformed before analysis. | | | | | | | | | | | | | | |  |
