# Supplementary material for: Association of skeletal muscle and serum metabolites with maximum power output gains in response to continuous endurance or high-intensity interval training programs: The TIMES study – A randomized controlled trial
Source: PLoS One. 2019 Feb 11;14(2):e0212115. doi: 10.1371/journal.pone.0212115 (PMC6370248; doi:10.1371/journal.pone.0212115)
Supplement: S4 Table — Data are mean ± standard deviation (SD) and skewness. ET: Continuous endurance training; HIIT: High-intensity interval training; MPO: Maximal power output; HRMAX: Maximal heart rate; ∆: Change Pre to Post intervention. * Difference from LRE (P < 0.0001 by unpaired t Test). (DOCX) [file pone.0212115.s011.docx]

| **S4 Table. Baseline characteristics of participants stratified by the 1^st^ and 3^rd^ tertiles of MPO (W) gains in response to ET and HIIT in TIMES.** | | | | | | | | | | | | | |
| --- | --- | --- | --- | --- | --- | --- | --- | --- | --- | --- | --- | --- | --- |
| **Variables** | **ET** | | | | | |  | **HIIT** | | | | | |
|  | **LRE (*n =* 10)** | | | **HRE (*n =* 10)** | | |  | **LRE (*n =* 10)** | | | **HRE (*n =* 10)** | | |
| Age (years) | 24.6 | ± | 3.2 | 22.5 | ± | 3.2 |  | 22.9 | ± | 2.3 | 23.5 | ± | 2.2 |
| Height (m) | 1.7 | ± | 0.0 | 1.8 | ± | 0.1 |  | 1.7 | ± | 0.1 | 1.8 | ± | 0.1 |
| Body mass (kg) | 69.5 | ± | 8.4 | 70.2 | ± | 13.7 |  | 73.0 | ± | 10.8 | 71.1 | ± | 11.2 |
| Body fat percentage (%) | 19.8 | ± | 5.6 | 16.4 | ± | 5.4 |  | 23.0 | ± | 9.7 | 20.9 | ± | 5.4 |
| BMI (kg m^2^) | 24.1 | ± | 2.1 | 22.6 | ± | 3.3 |  | 24.1 | ± | 2.6 | 23.1 | ± | 2.3 |
| MPO (W) | 255.9 | ± | 45.9 | 230.8 | ± | 28.4 |  | 229.0 | ± | 36.3 | 230.5 | ± | 28.9 |
| ∆ MPO (W) | 32.2 | ± | 8.6 | 65.8 | ± | 6.4* |  | 39.5 | ± | 6.0 | 76.9 | ± | 9.8* |
| ∆ MPO (%) | 13.1 | ± | 4.7 | 29.0 | ± | 5.1* |  | 17.7 | ± | 4.3 | 33.9 | ± | 6.3* |
| HR_MAX_ (beats min^-1^) | 195 | ± | 10 | 191 | ± | 6 |  | 187 | ± | 6 | 191 | ± | 8 |
| ∆ HR_MAX_ (beats min^-1^) | -3 | ± | 4 | 1 | ± | 4 |  | 0 | ± | 5 | 1 | ± | 3 |
| ∆ HR_MAX_ (%) | -1 | ± | 2 | 1 | ± | 2 |  | 0 | ± | 2 | 0 | ± | 2 |
| Data are mean ± standard deviation (SD) and skewness. ET: Continuous endurance training; HIIT: High-intensity interval training; MPO: Maximal power output; HR_MAX_: Maximal heart rate; ∆: Change Pre to Post intervention. * Difference from LRE (*P* < 0.0001 by unpaired *t* Test). | | | | | | | | | | | | | |
