# Supplementary material for: Association of skeletal muscle and serum metabolites with maximum power output gains in response to continuous endurance or high-intensity interval training programs: The TIMES study – A randomized controlled trial
Source: PLoS One. 2019 Feb 11;14(2):e0212115. doi: 10.1371/journal.pone.0212115 (PMC6370248; doi:10.1371/journal.pone.0212115)
Supplement: S5 Table — MPO: Maximal power output; ET: Continuous endurance training; HIIT: High-intensity interval training. (DOCX) [file pone.0212115.s012.docx]

| **S5 Table. Summary of serum and skeletal muscle metabolites supported by all 3 levels of evidence and related pathways associated to MPO gains in response to ET and HIIT in TIMES.** | | | | | | | | | | | | | | |
| --- | --- | --- | --- | --- | --- | --- | --- | --- | --- | --- | --- | --- | --- | --- |
| **Associated Pathways** | **ET** | | | | | | | |  | **HIIT** | | | | |
|  | **Serum** |  | **Skeletal muscle** | | | | | |  | **Serum** | | |  | **Skeletal mucsle** |
|  | **Glycerol** |  | **Pyruvate** | **Alanine** | **Proline** | **Threonine** | **Creatinine** | **AMP** |  | **Phenylalanine** | **Lysine** | **Creatine** |  | **Glycolate** |
| Alanine, aspartate and glutamate metabolism |  |  | X | X | X |  |  |  |  |  |  |  |  |  |
| Aminoacyl-tRNA biosynthesis |  |  |  | X | X | X |  |  |  | X | X |  |  |  |
| Arginine and proline metabolism |  |  |  |  | X |  | X |  |  |  |  | X |  |  |
| Citrate cycle |  |  | X |  |  |  |  |  |  |  |  |  |  |  |
| Glycerolipid metabolism | X |  |  |  |  |  |  |  |  |  |  |  |  |  |
| Glycine, serine and threonine metabolism |  |  | X |  |  | X |  |  |  |  |  | X |  |  |
| Glycolysis or gluconeogenesis |  |  | X |  |  |  |  |  |  |  |  |  |  |  |
| Glyoxylate and dicarboxylate metabolism |  |  | X |  |  |  |  |  |  |  |  |  |  | X |
| Nicotinate and nicotinamide metabolism |  |  | X |  |  |  |  |  |  |  |  |  |  |  |
| Nitrogen metabolism |  |  |  |  |  |  |  | X |  | X |  |  |  |  |
| Phenylalanine metabolism |  |  | X |  |  |  |  |  |  |  |  |  |  |  |
| Phenylalanine tyrosine and tryptophan biosynthesis |  |  |  |  |  |  |  |  |  | X |  |  |  |  |
| Purine metabolism |  |  |  |  |  |  |  | X |  |  |  |  |  |  |
| Pyruvate metabolism |  |  | X |  |  |  |  |  |  |  |  |  |  |  |
| Taurine and hypotaurine metabolism |  |  | X | X |  |  |  |  |  |  |  |  |  |  |
| Valine, leucine and isoleucine biosynthesis |  |  | X |  |  | X |  |  |  |  |  |  |  |  |
| MPO: Maximal power output; ET: Continuous endurance training; HIIT: High-intensity interval training. | | | | | | | | | | | | | | |
